# PI3 kinase inhibition improves vascular malformations in mouse models of hereditary haemorrhagic telangiectasia

**DOI:** 10.1038/ncomms13650

**Published:** 2016-11-29

**Authors:** Roxana Ola, Alexandre Dubrac, Jinah Han, Feng Zhang, Jennifer S. Fang, Bruno Larrivée, Monica Lee, Ana A. Urarte, Jan R. Kraehling, Gael Genet, Karen K. Hirschi, William C. Sessa, Francesc V. Canals, Mariona Graupera, Minhong Yan, Lawrence H. Young, Paul S. Oh, Anne Eichmann

**Affiliations:** 1 Cardiovascular Research Center, Department of Internal Medicine, Yale University School of Medicine, New Haven, Connecticut 06511, USA; 2 Department of Pharmacology, Yale University School of Medicine, New Haven, Connecticut 06520, USA; 3 Vascular Signalling Laboratory, Institut d'Investigació Biomèdica de Bellvitge, L'Hospitalet de Llobregat, Barcelona 08908, Spain; 4 Translation Research Laboratory, Catalan Institute of Oncology, Idibell, Barcelona 08908, Spain; 5 Molecular Oncology, Genentech, Inc., South San Francisco, California 94080-4990, USA; 6 Department of Physiology and Functional Genomics, University of Florida College of Medicine, PO Box 100274, Gainesville, Florida 32610, USA; 7 Department of Cellular and Molecular Physiology, Yale University School of Medicine, New Haven, Connecticut 06520, USA; 8 Inserm U970, Paris Cardiovascular Research Center, Paris 75015, France

## Abstract

Activin receptor-like kinase 1 (ALK1) is an endothelial serine–threonine kinase receptor for bone morphogenetic proteins (BMPs) 9 and 10. Inactivating mutations in the *ALK1* gene cause hereditary haemorrhagic telangiectasia type 2 (HHT2), a disabling disease characterized by excessive angiogenesis with arteriovenous malformations (AVMs). Here we show that inducible, endothelial-specific homozygous *Alk1* inactivation and BMP9/10 ligand blockade both lead to AVM formation in postnatal retinal vessels and internal organs including the gastrointestinal (GI) tract in mice. VEGF and PI3K/AKT signalling are increased on *Alk1* deletion and BMP9/10 ligand blockade. Genetic deletion of the signal-transducing *Vegfr2* receptor prevents excessive angiogenesis but does not fully revert AVM formation. In contrast, pharmacological PI3K inhibition efficiently prevents AVM formation and reverts established AVMs. Thus, *Alk1* deletion leads to increased endothelial PI3K pathway activation that may be a novel target for the treatment of vascular lesions in HHT2.

## Abstract (web summary)

Arteriovenous malformations (AVM) is a hallmark of hereditary haemorrhagic telangiectasia type 2, a disease caused by mutations in BMP receptor ALK1. Ola *et al*. show that AVM can be caused by blocking BMP9 and BMP10 in mice, leading to increased VEGF and PI3K activity, and that pharmacologic inhibition of PI3K prevents AVM development.

## Graphical abstract

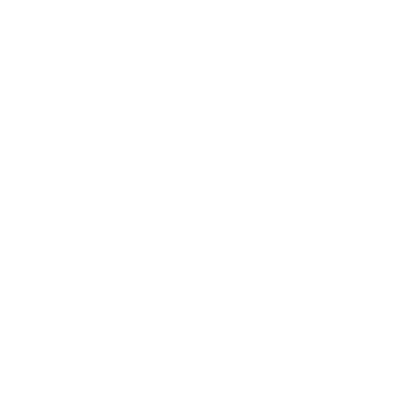

Haemorrhagic telangiectasia (HHT) is an autosomal-dominant inherited vascular disorder that affects ∼1 in 5,000 people. Patients develop multiple focal vascular malformations including capillary telangiectasies and arteriovenous malformations (AVMs)^1^. These lesions are fragile and prone to bleeding, and large calibre AVMs cause pulmonary and systemic shunting that can be physiologically significant^1^. More than 95% of HHT cases are caused by mutations in transforming growth factor-β (TGF-β)/bone morphogenetic protein (BMP) signalling pathway genes, including the surface receptors Endoglin (*ENG*, mutated in HHT1) and *ACVRL1* (hereafter referred as *ALK1*, mutated in HHT2), and the signalling pathway effector *SMAD4*-juvenile polyposis^2^,^3^,^4^,^5^. ENG and ALK1 proteins are expressed predominantly at the surface of endothelial cells, where they bind members of the TGF-β/BMP family, including TGF-β, BMP9 and BMP10 (refs 6, 7, 8). ENG acts as an auxiliary co-receptor that promotes signalling through ALK1 (ref. 9). Ligand binding activates cytoplasmic regulatory SMADs 1, 5 and 8, which subsequently complex with SMAD4 and translocate into the nucleus to regulate gene expression^7^. Thus, HHT is caused by mutations leading to deregulation in this signalling pathway, but how these mutations induce vascular malformations remains unclear. Identifying such mechanisms could provide novel approaches to prevent vascular malformations in HHT patients.

Mouse models have provided insights into *Eng* and *Alk1* function. Heterozygous mutations in either gene give rise to vascular lesions, but these form at low frequency and late in life, making them inconvenient to study^10^,^11^. Homozygous global *Eng*^10^ and *Alk1* gene inactivation in mice^6^ and zebrafish^12^ leads to embryonic death due to AVMs, again rendering the study of molecular mechanisms difficult. However, postnatal tamoxifen (Tx)-inducible, endothelial-specific homozygous deletion of either gene combined with angiogenic or pro-inflammatory stimuli induces HHT-like vascular malformations including excessive angiogenesis, enlarged veins and AVMs^13^,^14^,^15^,^16^. Thus, loss of both copies of endothelial *Alk1* or *Eng* genes in the context of active angiogenesis is thought to engender vascular lesions.

Here we investigate AVM formation in the retina and gastrointestinal (GI) tract using *Cdh5-Cre*^*ERT2*^ mice^17^ crossed with *Alk1*^fl/fl^ (*Alk1*^*iΔEC*^) mice^14^,^18^ in which endothelial-specific homozygous deletion of *Alk1* can be induced postnatally with Tx and blocking antibodies (blABs) against the BMP9/10 ligands^19^. We show that blocking BMP9-ALK1 signalling enhances pro-angiogenic signalling induced by vascular endothelial growth factor (VEGF), the major angiogenic growth factor known to date^20^, but also enhances phosphatidyl inositol 3-kinase (PI3K)-AKT signalling independently of exogenous VEGF. We show that targeting *Vegfr2* prevents angiogenesis in *Alk1*^*iΔEC*^ mice but does not rescue normal vascular patterning and AVM formation, whereas PI3K inhibition rescues vascular malformations in BMP signalling-deficient retinas and GI tract, identifying PI3K pathway inhibition as a novel putative treatment approach for HHT patients.

## Results

### Blocking BMP9/10 signalling leads to vascular malformations

**Figure 1:**
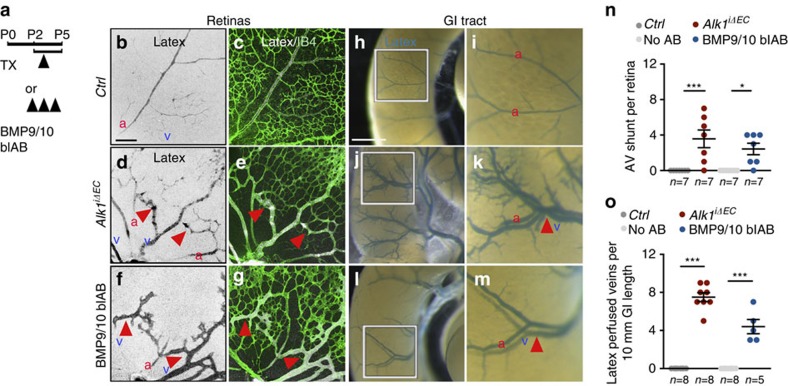
Blocking BMP9/10-Alk1 signalling induces vascular malformations. (**a**) Schematic representation of the experimental strategy used to delete *Alk1* or to block BMP9/10 ligands (P0–P5). Arrowheads indicate intra-gastric injection of 50 μg Tx at postnatal day 3 (P3) and i.p. administration of BMP9/10 blAB, (10 mg kg^−1^) at P2–P4. (**b**–**g**) P5 mouse retinas containing latex dye injected into the left ventricle of the heart. Vascular staining with latex dye (black in single images in **b**,**d**,**f** and white in merged images in **c**,**e**,**g**) and IB4, (green) in **c**,**e**,**g** of retinal flat mounts from control (*Ctrl*) (**b**,**c**), *Alk1*^*iΔEC*^ (**d**,**e**) and BMP9/10 blAB-injected (**f**,**g**) mice. Red arrowheads indicate retinal AVMs. (**h**–**m**) Vascular staining with latex dye of small intestine (blue) in *Ctrl* (**h**,**i**), *Alk1*^*iΔEC*^ (**j**,**k**) and BMP9/10 blAB-injected (**l**,**m**) P5 mice. **i**,**k**,**m** are magnified areas of boxed areas in **h**,**j**,**l**. Red arrowheads indicate latex-perfused veins. (**n**) Quantification of AV shunt number in the retinas. Each dot represents a retina. *n*=7 Retinas per group. Error bars: s.e.m., *P<0.05 and ***P<0.001, Mann–Whitney *U*-test. (**o**) Quantification of latex-perfused veins at the surface of the small intestine. Each dot represents a perfused vein. *n*=5–8 intestines. Error bars: s.e.m., ***P<0.001, Mann–Whitney *U*-test. Scale bars, 100 μm in **b**–**g** and 10 mm in **h**,**j**,**l**. a, artery in red; v, vein in blue.

We used postnatal day 5 (P5) *Cdh5-Cre*^*ERT2*^;*Alk1*^fl/fl^ mice (*Alk1*^*iΔEC*^) treated with a single dose of 50 μg Tx at P3 and mice treated with BMP9/10 blAB^19^ (10 mg kg^−1^, intraperitoneal (i.p.)) between P2 and P4 (Fig. 1a and Supplementary Fig. 1a). Tx-injected Cre-negative littermates and uninjected wild-type (no antibody) littermates were used as controls. Efficient *Alk1* gene deletion was verified by quantitative PCR (qPCR) from mouse lung endothelial cells (mLECs; Supplementary Fig. 1b) and loss of endothelial staining with an anti-Alk1 antibody (Supplementary Fig. 1c,d). BMP9/10 blockade did not affect Alk1 expression (Supplementary Fig. 1c,e).

At P5, the morphology of retinal and organ vasculature was analysed by injection of latex dye (Fig. 1). Control retinas and the GI tract showed staining of arterial but not venous vessels, because latex cannot perfuse capillaries (Fig. 1b,c,h,i). In contrast, both *Alk1*^*iΔEC*^- and BMP9/10 blAB-treated mice exhibited latex dye staining of veins in retinas (Fig. 1d–g) and the GI tract (Fig. 1j–m). Counterstaining of retinal vessels with IsolectinB4 (IB4) showed that latex perfused AVMs with blood flowing directly from an artery into a vein without an intervening capillary bed (Fig. 1d–g). Quantification revealed an average of three latex-perfused AVMs per retina in *Alk1*^*iΔEC*^- and two AVMs in BMP9/10 antibody-treated mice (Fig. 1n). The GI tract surface of mutants showed an average of seven in *Alk1*^*iΔEC*^ and four in BMP9/10 blAB perfused veins per 10 mm gut length, whereas no perfused veins were seen in control mice (Fig. 1o).

Additional analysis of IB4-stained retinas confirmed previous results^15^,^21^ that AVMs were usually located in the centre of the retina, close to the optic nerve, whereas increased vessel area and branching were observed at the vascular front in both *Alk1*^*iΔEC*^- and BMP9/10 blAB-treated mice (Supplementary Fig. 1f–k).

Sorting of endothelial cells from *Alk1*^*iΔEC*^ retinas showed that *Alk1*-deficient cells display enhanced cell cycle progression, with a significant reduction of cells in G1 phase and a concomitant increase of endothelial cells in S/G2/M (Supplementary Fig. 2a). Staining of BMP signalling-deficient retinas for smooth muscle actin (SMA) showed a decrease in arterial and an increase in venous SMA coverage when compared with control retinas, in particular in veins engaged in AVMs (Supplementary Fig. 2b–d). qPCR analysis on mLECs isolated from *Alk1*^*iΔEC*^ showed unchanged levels of *Vegfr2* but downregulation of *Alk1*, *Eng* and *Vegfr1*, as well as of Notch signalling components *Notch1* and *Jag1* (Supplementary Fig. 2e), in agreement with a previous report^15^. Arterial markers *Unc5b* and *Efnb2* (refs 17, 22) were decreased, whereas expression of the venous marker *EphB4* was upregulated in *Alk1* mutant cells (Supplementary Fig. 2e). Antibody staining confirmed that *Alk1* mutant cells lose expression of Vegfr1 (Supplementary Fig. 2f,g), a negative regulator of VEGF signalling, and of the Notch ligand Jagged 1 (Supplementary Fig. 2h,i)^20^,^23^, suggesting that reduced Notch and enhanced VEGF signalling could contribute to AVM formation in *Alk1* mutant cells.

### *Vegfr2* deletion prevents vascular development in *Alk1* mutants

**Figure 2:**
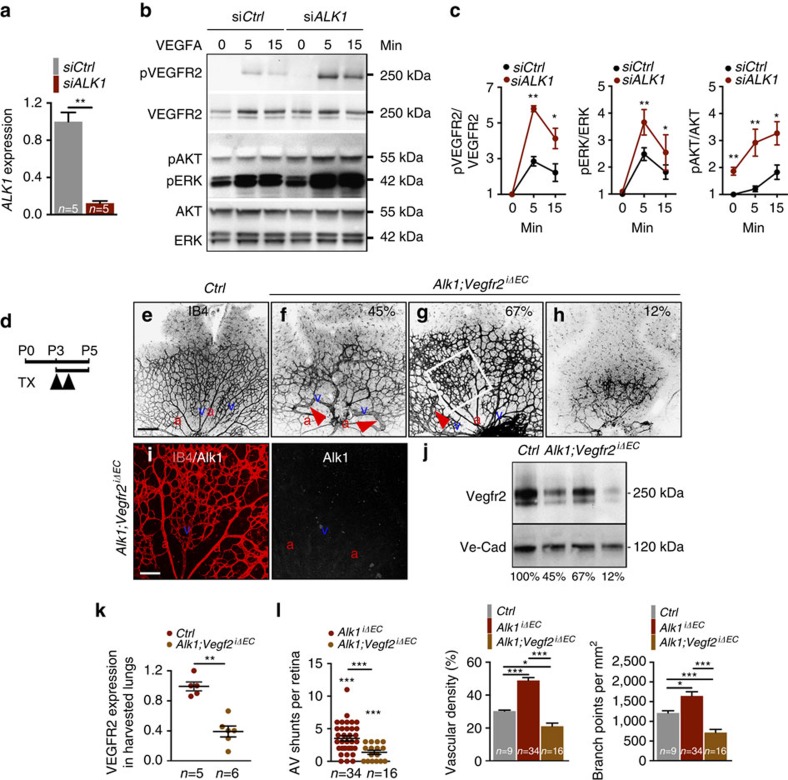
*Vegfr2* deletion blocks angiogenesis in *Alk1*^*iΔEC*^ mice. (**a**) *ALK1* expression analysis by qPCR in HUVECs transfected with *Ctrl* or *ALK1* siRNA. (**b**) Western blot analysis for VEGFR2, AKT and ERK activation in HUVECs transfected with *Ctrl* or *ALK1* siRNA and treated with 10 ng VEGFA for 0–15 min. (**c**) Quantification of western blottings corresponding to **b** (*n*=5 independent experiments). Error bars: s.e.m., *P<0.05 and **P<0.01, Mann–Whitney *U*-test. (**d**) Schematic representation of the experimental strategy to assess vasculature in *Alk1;Vegfr2*^*iΔEC*^ P5 mice. Arrowheads indicate intra-gastric injections of 100 μg Tx at P3 and P4. (**e**–**h**) IB4 staining of retinal flat mounts (negative images of the fluorescent signal) from *Ctrl* mice (**e**) and three different *Alk1;Vegfr2*^*iΔEC*^ P5 mice (**f**–**h**). Red arrowheads in **f**,**g** indicate AV shunts. (**i**) IB4 and Alk1 double labelling of the *Alk1;Vegfr2*^*iΔEC*^ retina shown in **g** (white square). (**j**) Vegfr2 protein expression in P5 lung lysates harvested from the mice shown in **e**–**h**. Percentage of residual Vegfr2 expression in each lysate is indicated in the corresponding panels in **f**–**h** and below the blot. (**k**) Quantification of Vegfr2 protein normalized to Ve-Cadherin detected by western blotting shown in **j**. Each dot represents 1 lung. *n*=5–6 mice per group. Error bars: s.e.m., **P<0.01, Mann–Whitney *U*-test. (**l**) Quantification of AV shunt number, vascular density and number of branch points. Each dot represents one retina. *n*=9–34 Retinas per group. Error bars: s.e.m., *P<0.05 and ***P<0.001, Mann–Whitney *U*-test. Scale bars, 200 μm in **e**–**h** and 100 μm in **i**. a, artery in red; v, vein in blue.

To study VEGF signalling in *ALK1*-deficient cells, we transfected human umbilical vein endothelial cells (HUVECs) with *ALK1* small interfering RNA (siRNA), which decreased *ALK1* messenger RNA levels by >90% (Fig. 2a). Compared with *Ctrl* siRNA, *ALK1*-depleted cells displayed equal levels of total VEGFR2 but showed an increase in VEGFR2 phosphorylation and activation of downstream pERK and pAKT in response to 10 ng VEGFA (Fig. 2b,c). Of note, pAKT was already increased at baseline in *ALK1* knockdown cells (Fig. 2b,c) in the absence of exogenous VEGF.

We reasoned that blocking the Vegf signal-transducing receptor Vegfr2 (ref. 20) might prevent vascular malformations in *Alk1* mutant retinal vessels. To block *Vegfr2* genetically, we used *Vegfr2*^fl/fl^ mice^24^ intercrossed with *Alk1*^*iΔEC*^ mice. One hundred micrograms of Tx was administered at P3 and P4, and *Alk1;Vegfr2*^*iΔEC*^ mice were analysed at P5 (Fig. 2d). Tx-injected Cre-negative littermates were used as controls (Fig. 2d,e). Compared with control mice, combined deletion of both *Vegfr2* and *Alk1* produced varying degrees of vascular defects that correlated with the extent of *Vegfr2* deletion (Fig. 2e–k). *Alk1;Vegfr2*^*iΔEC*^ mice had a reduced number of AVMs when compared with *Alk1*^*iΔEC*^ single mutants, but many retinas still exhibited AVMs (Fig. 2f,l), despite loss of Alk1 expression (Fig. 2i).

Western blot analysis of Vegfr2 expression (Fig. 2j) in the lungs corresponding to the retinas in Fig. 2e–h showed that mice with inefficient *Vegfr2* deletion (33%) could display close to normal retina vascular patterning (Fig. 2g,i). In contrast, mice with efficient *Vegfr2* deletion (88%) had strongly reduced retinal angiogenesis, in some cases displaying barely any vasculature (Fig. 2h). Quantification of 16 retinas showed that overall vascular density and branching in double mutants was significantly lower than untreated controls (Fig. 2l), indicating that Vegfr2 inhibition was dominant over *Alk1* loss of function and, if complete, prevented retinal vascular development.

### Increased PI3K signalling in Alk1-deficient endothelial cells

**Figure 3:**
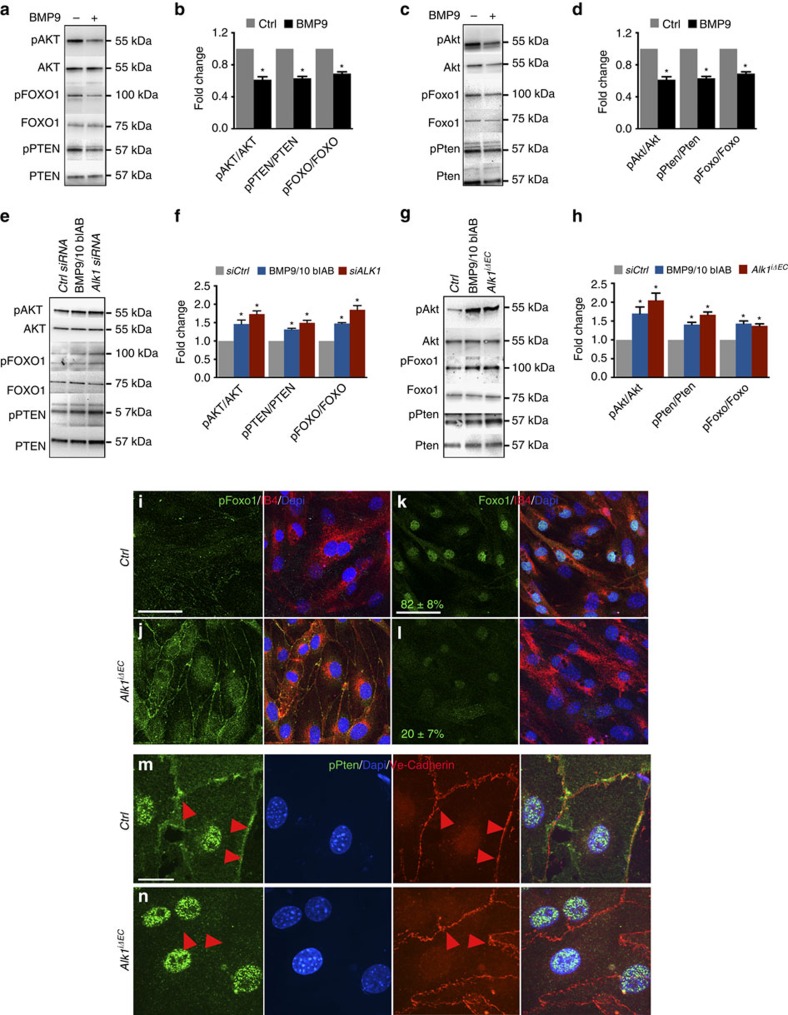
*Alk1* deletion increases PI3K signalling. (**a**,**c**) Western blot (Wb) analysis of HUVECs (**a**) and mLECs (**c**) stimulated or not with BMP9 for 2 h. (**b**,**d**) Quantifications of HUVECs (**b**) and mLECs (**d**) pAKT, pPTEN and pFOXO1 levels normalized to total AKT, PTEN and FOXO1, respectively. (**e**) Wb analysis of HUVECs transfected with *Ctrl* or *ALK1* siRNA or treated with 1 μg ml^−1^ of BMP9/10 blAB for 36 h. (**f**) Quantifications of pAKT, pPTEN and pFOXO1 levels normalized to total AKT, PTEN and FOXO1, respectively. (**g**) Wb analysis of mLECs with the indicated genotype or treated with 1 μg ml^−1^ of BMP9/10 blAB for 36 h. (**h**) Quantifications of pAkt, pFoxo1 and pPten levels normalized to total Akt, Foxo1 and Pten, respectively. Graphs represent mean of *n*=4 experiments. Error bars: s.e.m., *P<0.05, Student's *T*-test. (**i**,**j**), pFoxo1 (green), IB4 (red) and Dapi (blue) staining of *Ctrl* (**i**) and *Alk1*^*iΔEC*^ (**j**) mLECs in culture. (**k**,**l**) Foxo1 (green), IB4 (red) and Dapi (blue) staining for *Ctrl* (**k**) or *Alk1*^*iΔEC*^ (**l**) mLECs. Numbers represent percentage of cells with nuclear Foxo1 staining. (**m**,**n**) Anti-pPten (green), Dapi (blue) and Ve-Cadherin (red) staining for *Ctrl* (**m**) and *Alk1*^*iΔEC*^ (**n**) mLECs in culture. Red arrowheads indicate pPten expression at plasma membrane. Scale bars, 100 μm in **i**–**l** and 10 μm in **m**,**n**.

As *ALK1* knockdown HUVECs exhibited increased AKT phosphorylation in the absence of VEGF stimulation (Fig. 2b,c), we investigated activation of the PI3K/AKT pathway in HUVECs (Fig. 3a,b) and mLECs (Fig. 3c,d) treated with BMP9. Two hours after treatment, AKT phosphorylation at serine 473 and phosphorylation of downstream FOXO1 were both strongly decreased compared with untreated cells (Fig. 3a–d). Upstream of PI3K-AKT, we found that phosphorylation of the Ser380/Thr382/Thr383-inactive form of the lipid phosphatase and tensin homologue (PTEN)^25^,^26^ was decreased in BMP9-stimulated HUVECs and mLECs (Fig. 3a–d), suggesting that AKT activity might be repressed by stabilizing PTEN at the cell membrane. Total PTEN levels were also decreased by BMP9 treatment (Fig. 3a,c). We next confirmed that BMP9/ALK1 signalling regulates AKT activation by using *ALK1*-depleted HUVECs and mLECs or cells treated with anti-BMP9/10 blABs (Fig. 3e–h). Cells were cultured in medium complemented with 2% fetal calf serum containing BMP9 proteins^21^. *ALK1*-depleted cells and BMP ligand blockade both increased pAKT, pFOXO1 and pPTEN levels (Fig. 3e–h).

In line with the western blotting results, immunostaining of *Alk1*^*iΔEC*^ mLECs showed an increase in PI3K-Akt signalling. Compared with control cells, pFoxo1 was increased in *Alk1*^*iΔEC*^ cells (Fig. 3i,j), whereas nuclear Foxo1 staining, which is inhibited by phosphorylation^27^, was decreased (Fig. 3k,l). pPten immunostaining in *Ctrl* mLECs revealed both nuclear and membrane staining, whereas we observed an increase in nuclear and a decrease in membrane staining in *Alk1*^*iΔEC*^ cells (Fig. 3m,n), suggesting that BMP inhibits PI3K-Akt activity via regulation of Pten localization and activity^25^,^26^.

### PI3K inhibition improves AVMs in *Alk1* mutants

**Figure 4:**
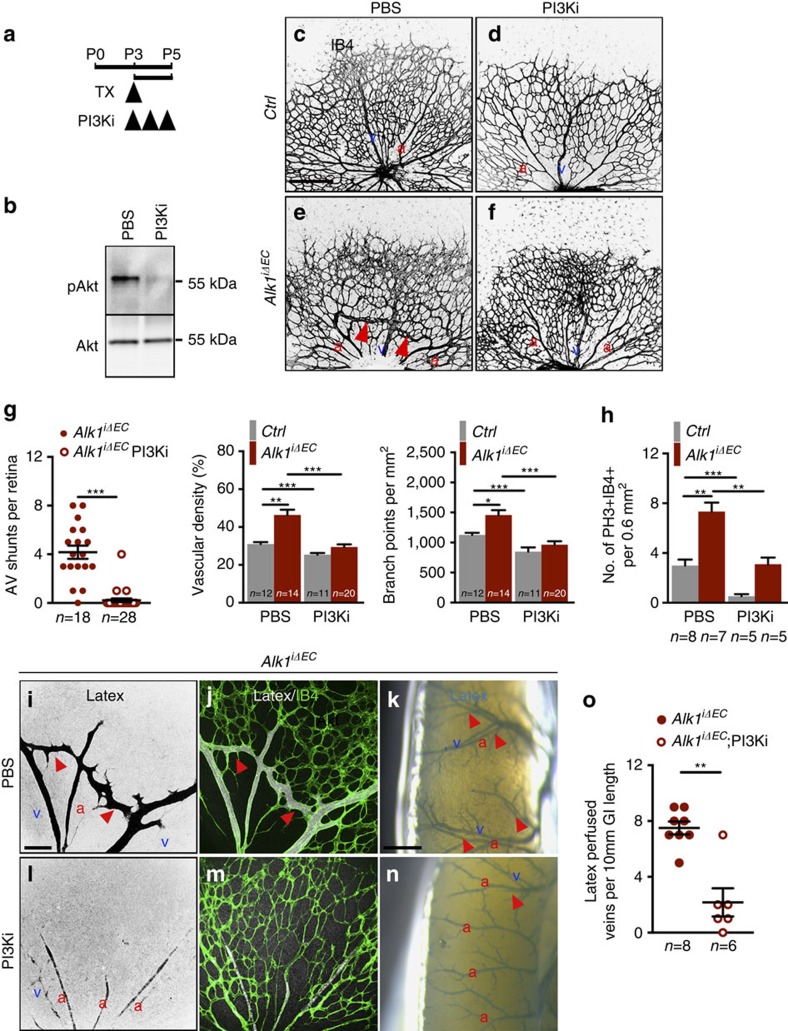
PI3K inhibition prevents vascular defects in *Alk1*^*iΔEC*^ mice. (**a**) Schematic representation of the experimental strategy to assess the effects of PI3K inhibition on *Alk1*^*iΔEC*^ vasculature. Arrowheads indicate the time course of Tx (50 μg) and Wortmannin (PI3Ki, 0.4 mg kg^−1^) or PBS (vehicle) administration. (**b**) Western blot analysis of Akt activation (pAkt) in total lung lysates from PBS or PI3K inhibitor-treated wild-type mice. (**c**–**f**) IB4 staining of retinal flat mounts (negative images of the fluorescent signal) from *Ctrl* (**c**,**d**) and *Alk1*^*iΔEC*^ P5 mice (**e**,**f**) injected with vehicle (**c**,**e**) or PI3Ki (**d**,**f**). Red arrowheads indicate AV shunts. (**g**) Quantification of the AV shunt number, vascular density and number of branchpoints. Each dot represents one retina. *n*=11–28 retinas per group. Error bars: s.e.m., *P<0.05, **P<0.01 and ***P<0.001, Mann–Whitney *U*-test. (**h**) Quantification of PH3+, IB4+ endothelial cells of P5 retinas harvested from mice with the indicated genotypes treated with PBS or PI3Ki. *n*=5–8 retinas per group. Error bars: s.e.m., **P<0.01 and ***P<0.001, Mann–Whitney *U*-test. (**i**,**j**,**l**,**m**) Vascular staining with latex dye (black in single images in **i**,**l** and white in merged images in **j**,**m**) and IB4 (green) of retinal flat mounts from *Alk1*^*iΔEC*^ P5 mice treated with PBS (**i**,**j**) or PI3Ki (**l**,**m**). (**k**,**n**) Vascular staining of the small intestine surface (blue) of *Alk1*^*iΔEC*^ treated with PBS (**k**) or PI3Ki (**n**) P5 mice. Red arrowheads in **i**–**k**,**n** indicate latex-perfused veins. (**o**) Quantification of latex-perfused veins. Each dot represents one intestine. *n*=6–8 Intestines per group. Error bars: s.e.m., **P<0.01, Mann–Whitney *U*-test. Scale bars, 200 μm in **c**–**f**, 100 μm in **i**,**l** and 500 μm in **k**,**n**. a, artery in red; v, vein in blue.

The data suggested that inhibition of PI3K signalling might improve vascular malformations in *Alk1*^*iΔEC*^ mice. To test this hypothesis, we injected 50 μg Tx into P3 *Alk1*^*iΔEC*^ mice and administered Wortmannin (PI3Ki, 0.4 mg kg^−1^, i.p.) at P3, P4 and P5 (Fig. 4a). Tx-injected Cre-negative littermates were used as controls and retinas were analysed at P5. Western blotting on whole lung lysates showed that PI3Ki efficiently inhibited AKT phosphorylation, without affecting total AKT levels (Fig. 4b). PI3Ki induced a decrease of retinal vasculature density and the number of branch points when compared with untreated *Ctrl* mice (Fig. 4c,d). PI3Ki also efficiently normalized the vasculature of *Alk1* mutants (Fig. 4f compared with Fig. 4e). Quantification showed that PI3Ki treatment prevented AV shunt formation in *Alk1*^*iΔEC*^ retinas and normalized vessel density, as well as the number of branch points close to control levels (Fig. 4g). We also found that PI3K inhibition in *Alk1*^*iΔEC*^ normalized the increased proliferation of the endothelial cells, which was assessed by PH3 staining (Fig. 4h).

We next tested the efficiency of PI3Ki in rescuing AVM formation in the internal organs. In latex-perfused control versus *Alk1*^*iΔEC*^ mice, PI3Ki efficiently reduced the number of latex-perfused retinal AVMs and veins on the surface of the GI tracts (Fig. 4i–o).

To confirm the effects of PI3K pathway inhibition in rescuing AVMs in *Alk1*^*iΔEC*^ mice we used another PI3K inhibitor, Pictilisib (PI3Ki 2)^28^. *Alk1* deletion was induced at P3 with 50 μg Tx and 20 mg kg^−1^ of PI3Ki2 was administered i.p. at P3–P5 and retinal vasculature was analysed at P5 (Supplementary Fig. 3a). Staining for IB4 shows that inhibition of PI3K activity with PI3Ki2 efficiently prevented the development of AV shunts and rescued vascular defects in *Alk1*^*iΔEC*^ retinas (Supplementary Fig. 3b–d).

**Figure 5:**
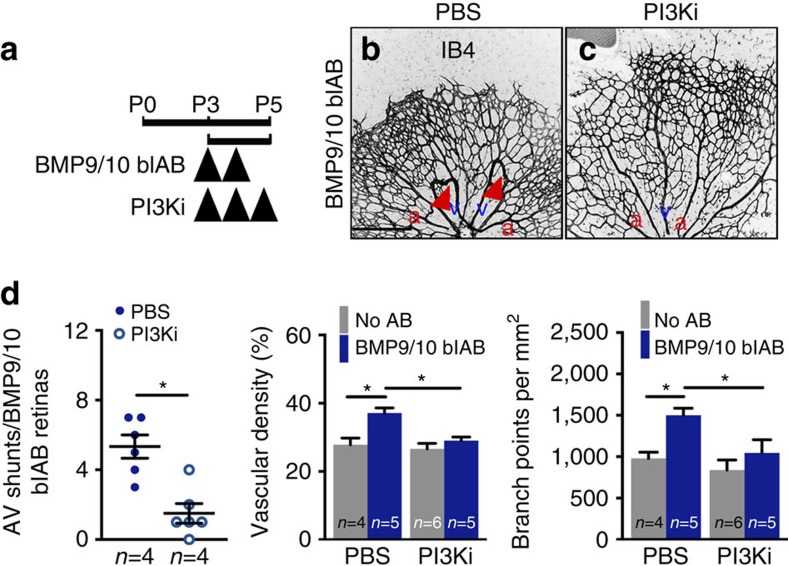
PI3K inhibition prevents AVM formation in BMP9/10 blAB-treated mice. (**a**) Schematic representation of the experimental strategy to assess the effects of PI3K inhibition on the retinal vasculature of BMP9/10 blAB injected mice. Arrowheads indicate the time course of injection of BMP9/10 blAB and PI3Ki or PBS. (**b**,**c**) IB4 staining of retinal flat mounts from mice treated with BMP9/10 blAB (**b**) and BMP9/10 blAB and PI3Ki (**c**). Red arrowheads in **b** indicate AV shunts. Scale bars, 200 μm in **b**,**c**. (**d**) Quantification of AV shunt number, vascular density and number of branch points. *n*=4–6 Retinas per group. Each dot represents one retina. Error bars: s.e.m., *P<0.05, Mann–Whitney *U*-test. a, artery in red; v, vein in blue.

PI3Ki treatment also prevented vascular defects induced by BMP9/10 blockade (Fig. 5a–d). BMP9/10 blAB and PI3Ki were administered at P3 and P4, and P3–P5, respectively, and the retinal vasculature was analysed at P5 (Fig. 5a). Staining for IB4 showed that inhibition of PI3K activity efficiently prevented the development of AV shunts and rescued vascular defects induced by BMP9/10 blAB (Fig. 5b–d).

**Figure 6:**
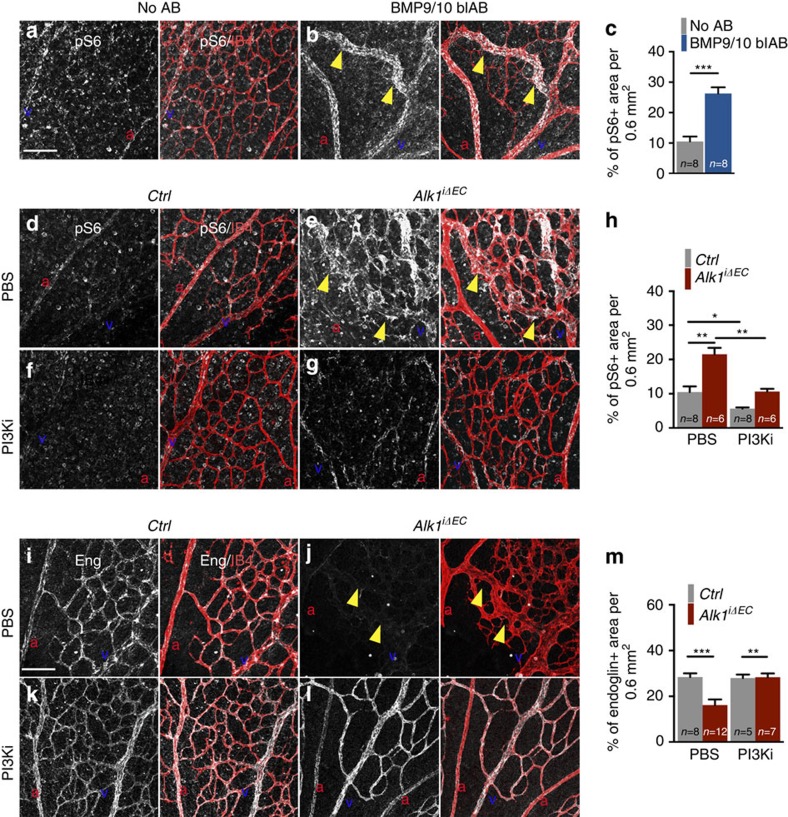
PI3K inhibition rescues downstream signalling components. (**a**,**b**) Anti phospho-S6 (pS6) and IB4 staining of retinal flat mounts from P5 mice treated or not with BMP9/10 blAB. (**c**) Quantification of the percentage of pS6+ vascular area per field of view. *n*=8 Retinas per group. Error bars: s.e.m., ***P<0.001, Mann–Whitney *U*-test. (**d**–**g**) Anti-pS6 and IB4 staining of retinal flat mounts from *Ctrl* (**d**,**f**) or *Alk1*^*iΔEC*^ (**e**,**g**) treated with PBS (**d**,**e**) or PI3Ki (**f**,**g**). (**h**) Quantification of the percentage of pS6+ vascular area per field of view. *n*=6–8 Retinas per group. Error bars: s.e.m., *P<0.05 and **P<0.01, Mann–Whitney *U*-test. (**i**,**l**) Anti-Eng and IB4 staining of retinal flat mounts from *Ctrl* (**i**,**k**) or *Alk1*^*iΔEC*^ (**j**,**l**) treated with PBS (**i**,**j**) or PI3Ki (**k**,**l**). (**m**) Quantification of the percentage of Eng+ vascular area per field of view. Yellow arrowheads in **b**,**e**,**j** indicate AV shunts. *n*=5–12 Retinas per group. Error bars: s.e.m., **P<0.01 and ***P<0.001, Mann–Whitney *U*-test. Scale bars, 100 μm in **a**,**b**,**d**–**g**,**i**–**l**. a, artery in red; v, vein in blue.

To confirm the efficacy of PI3K inhibition *in vivo*, treated versus untreated controls, versus *Alk1*^*iΔEC*^ and BMP9/10 blAB-injected mice retinas were labelled for PI3K downstream signalling components (Fig. 6a–m). Endothelial nuclear Foxo1 staining^29^ was strongly decreased in *Alk1* mutant and BMP9/10 blAB-treated mice (Supplementary Fig. 4a–d). Staining with phospho-S6 (ref. 30), which is positively regulated by PI3K-AKT signalling was strongly increased in BMP9/10 blAB- (Fig. 6b,c) and *Alk1*^*iΔEC*^*-*treated retinas (Fig. 6e,h) when compared with control retinas (Fig. 6a,d). PI3Ki treatment decreased the expression of phospho-S6 in controls (Fig. 6f,h) and in *Alk1* mutant retinas (Fig. 6g,h). Furthermore, Eng, which is induced by Alk1 signalling^15^ and repressed by PI3K (ref. 31), was decreased in *Alk1* mutant retinal vessels (Fig. 6j,m) when compared with control retinas (Fig. 6i,m) and the expression was rescued by PI3Ki treatment (Fig. 6l,m).

### PI3K inhibition reverts established AVMs in Alk1 mutants

**Figure 7:**
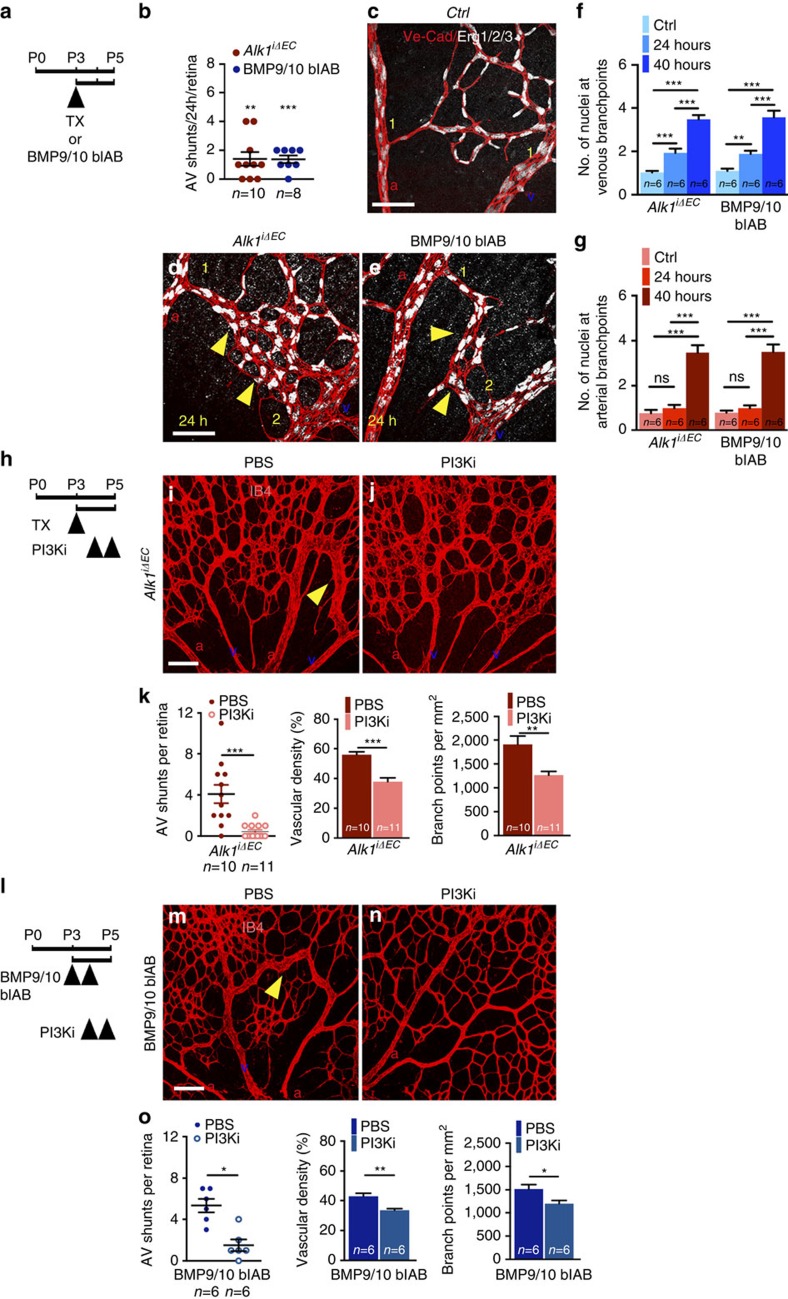
PI3K inhibition rescues established retinal AVMs. (**a**) Schematic representation of the experimental strategy to assess *Alk1* deletion or BMP9/10 blockade. Arrowhead indicates injection of 50 μg Tx or BMP9/10 blAB. (**b**) Quantification of AV shunt number 24 h after Tx or BMP9/10 blAB administration. *n*=8–10 Retinas per group. Error bars: s.e.m. **P<0.01 and ***P<0.001, Mann–Whitney *U*-test. (**c**–**e**) Ve-Cadherin and Erg1/2/3 staining of retinal flat mounts from *Ctrl* (**c**), *Alk1*^*iΔEC*^- (**d**) and BMP9/10 blAB- (**e**) treated P4 mice. Yellow arrowheads in **d**,**e** indicate AV shunts. Numbers of ERG1/2/3+ endothelial cell nuclei at branches of retinal veins and arteries are indicated in yellow. (**f**,**g**) Quantification of the number of nuclei at the connection of capillaries with veins (**f**) or arteries (**g**) in P4 and P5 retinal vessels. *n*=6 retinas per group. Error bars: s.e.m., **P<0.01 and ***P<0.001, Mann–Whitney *U*-test. (**h**) Schematic representation of the experimental strategy to assess the effects of PI3K inhibition on *Alk1*^iΔEC^ vasculature 24 h post Tx injection. Arrowheads indicate the time course of Tx (50 μg) and PI3Ki injections. (**i**,**j**) IB4 staining of retinal flat mounts from *Alk1*^iΔEC^ P5 mice treated with PBS (**i**) or PI3Ki 24 h post Tx injection (**j**). Yellow arrowheads in **i**,**m** indicate AV shunts. (**k**) Quantification of AV shunt number, vascular density and number of branchpoints. *n*=10–11 Retinas per group. Error bars: s.e.m. **P<0.01 and ***P<0.001, Mann–Whitney *U*-test. (**l**) Schematic representation of the experimental strategy to assess the effects of PI3K inhibition on the retinal vasculature 24 h post BMP9/10 blAB-treated mice. Arrowheads indicate the time course of BMP9/10 blAB and PI3Ki administration. (**m**,**n**) IB4 staining of retinal flat mounts from mice treated with BMP9/10 blAB and 24 h later with PBS (**m**) or PI3Ki (**n**). Yellow arrowheads indicate AV shunts. (**o**) Quantification of AV shunt number, vascular density and number of branch points. *n*=6 Retinas per group. Error bars: s.e.m., *P<0.05 and **P<0.01, Mann–Whitney *U*-test. Scale bars, 100 μm in **c**–**e**,**i**,**j**,**m**,**n**. a, artery in red; v, vein in blue.

To address whether inhibition of PI3K was able to revert already established AVMs, we first determined the time course of AVM formation in *Alk1*^*iΔEC*^- and BMP9/10 blAB-treated retinas. Fifty micrograms of Tx or BMP9/10 blABs were administered at P3 in *Alk1*^*iΔEC*^ or control pups, respectively, and retinas were analysed 24 (P4) or 40 h later (P5) (Fig. 7a). Seventy per cent of *Alk1*^*iΔEC*^ retinas and 87% of BMP9/10 blAB-treated retinas exhibited at least one AV shunt 24 h after injection (Fig. 7b). Staining for the endothelial nuclear Erg1,2,3 transcription factor^32^ and Ve-Cadherin showed an increase in the number of endothelial cells specifically at branches of retinal veins (Fig. 7c–g). In control retinas, these branches contained a single cell (Fig. 7c), whereas *Alk1*^*iΔEC*^ or BMP9/10 blAB-treated mice showed two to three cells (Fig. 7d–f). At 40 h, both arterial and venous branches in mutant AVMs exhibited more than three cells compared with one cell in controls (Fig. 7c–g), suggesting that AVMs arise from the enlargement of capillaries and an increase in the cell number at venous and arterial branch points.

Having established that AVMs were present at P4, we next induced gene deletion with 50 μg Tx at P3 and administered PI3Ki 24 and 40 h later. Pups were killed at P5 (Fig. 7h). Analysis of retinal vasculature showed that PI3Ki efficiently rescued vascular anomalies in *Alk1*^*iΔEC*^ retinas (Fig. 7i–k). Likewise, PI3Ki treatment at P4 after induction of BMP9/10 blockade at P3 and P4 also reduced vascular defects (Fig. 7l–o), suggesting that inhibition of PI3K pathway reverts already established AVMs.

## Discussion

**Figure 8:**
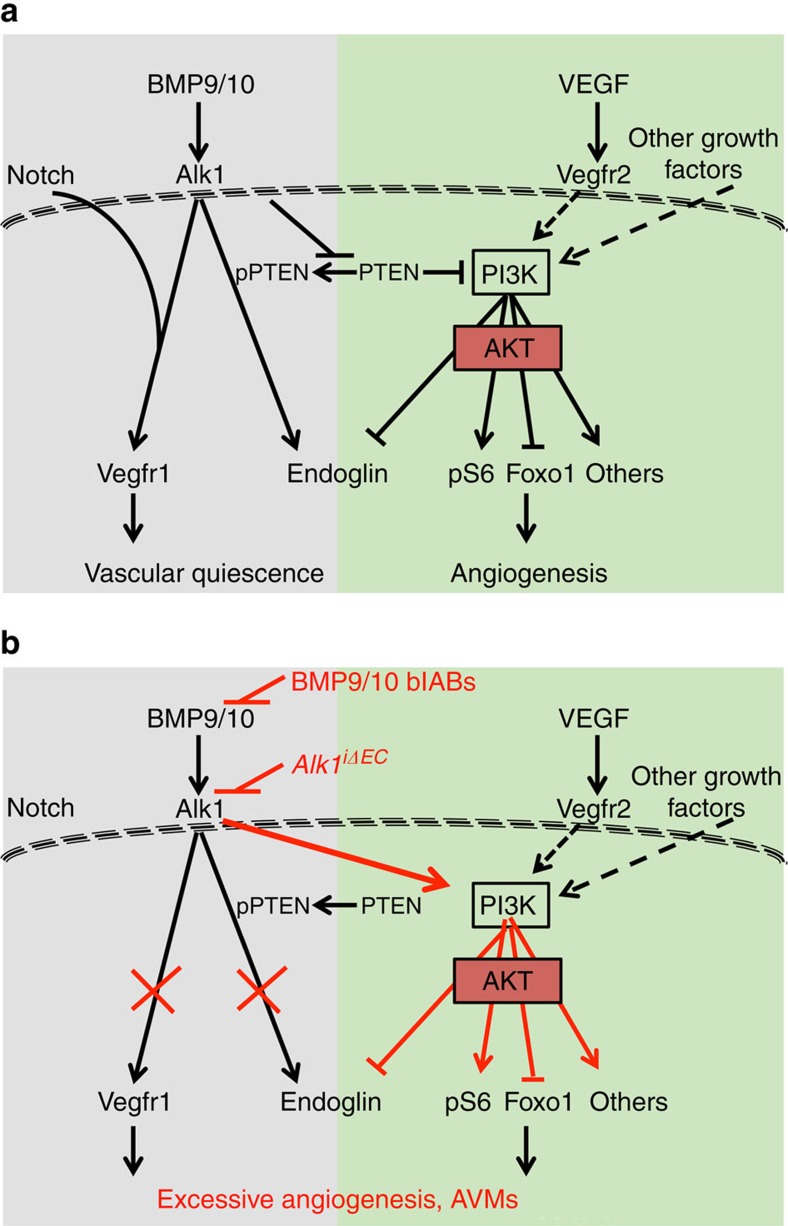
Model for BMP9/10 signalling in maintenance of vascular quiescence. (**a**) BMP9/10 signalling through the Alk1 receptor in endothelial cells synergizes with Notch to induce expression of the anti-angiogenic receptor –Vegfr1 thereby repressing Vegf signalling and angiogenesis. Alk1 signalling also represses PI3K activation downstream of Vegfr2 and other growth factors, through inhibition of Pten expression and phosphorylation. PI3K promotes angiogenesis via Akt and downstream activation of ribosomal S6 (pS6), inhibition of Foxo1 and regulation of other downstream effectors. (**b**) Blocking BMP9/10-Alk1 signalling results in increased Pten expression and phosphorylation, and consequently in an excessive PI3K signalling, thereby inducing vascular defects. Blocking PI3K with PI3K inhibitors rescues excessive angiogenesis and vascular malformations in BMP signalling deficient mice.

The major novel finding reported here is that inhibition of BMP9 and 10 signalling through the Alk1 receptor leads to overactivation of the PI3K-Akt pathway and that inhibition of PI3K activity rescues vascular malformations in mouse models of HHT2 (Fig. 8). The PI3K/Akt pathway stimulates endothelial cell proliferation, migration and survival downstream of various angiogenic growth factors^33^. We show that pharmacological inhibition of PI3K signalling using two different inhibitors prevents and even rescues abnormal features in BMP signalling-deficient retinas and the GI tract. Inhibition of PI3K in wild-type retinas closely mimics the retinal angiogenesis phenotype in mice lacking the *PI3K110α* isoform or *Akt1* gene^34^,^35^ confirming treatment specificity. However, PI3K inhibition rescued excessive retinal endothelial cell proliferation, providing one possible mechanism for its effects *in vivo*. In addition, a recent study showed that AVM formation in zebrafish *Alk1* mutants was due to altered endothelial cell migration^36^, which in mouse retinas is dependent on the PI3K110α isoform^34^. Therefore, with increased endothelial cell proliferation, altered migration is likely to be implicated in the *Alk1* mutant phenotype and may be targeted by PI3K inhibition. This pathway therefore represents an attractive potential therapy for Alk1-mediated HHT2 disease.

Mechanistically, we found that enhanced PI3K-Akt pathway activation following BMP9 signalling blockade involves the upstream lipid phosphatase Pten (Fig. 8). BMP9 was previously shown to decrease Pten expression levels in mouse embryonic fibroblasts^37^, which prompted us to investigate Pten as a possible upstream regulator of PI3K-Akt phosphorylation. We confirmed changes in total Pten levels and additionally found more dramatic effects on the C-terminal Pten phosphorylation. Pten catalyses the conversion of phosphatidylinositol 3,4,5-trisphosphate into phosphatidylinositol 4,5-bisphosphate, thereby counteracting PI3K-Akt signalling at the cell membrane. When phosphorylated, Pten assumes a closed conformation with an inactive phosphatase domain that is unable to bind the cell membrane and to dephosphorylate PI3K substrate, thereby increasing PI3K activity^25^,^38^.

Our *in vitro* experiments show that BMP9 treatment decreases carboxy-terminal PTEN phosphorylation in HUVECs and mLECs, thereby increasing PTEN activity at the cell membrane, leading to reduced phosphorylation of AKT and of downstream FOXO1 transcription factor. Conversely, loss-of-function experiments using *Alk1* knockout mLECs or knockdown HUVECs, or BMP9/10 blAB-treated mLECs and HUVECs show the reverse effect: increased PTEN phosphorylation led to increased AKT and FOXO1 phosphorylation. This points to regulation of C-terminal PTEN phosphorylation by BMP9 as the critical step for AKT activation (Fig. 8). We are currently investigating the mechanistic basis for this effect. BMP9/ALK1 might signal via SMAD proteins to repress transcription of PTEN and/or induce kinases targeting the PTEN C terminus.

*PTEN* mutations in humans cause vascular anomalies^39^, suggesting PTEN as a major hub to maintain the proper balance between BMP and PI3K signalling pathways. Likewise, overactivation of the PI3K/AKT/mammalian target of rapamycin pathway has been shown to cause vascular anomalies. Activating mutations of *AKT*/Protein kinase B (ref. 40), *TIE2* receptor (ref. 41) or *PIK3CA* (ref. 42) have been associated with the development of vascular anomalies in mice and humans. Interestingly, the absence of AV shunts in these pathologies, or in *Pten*-deficient mice retinas suggests that an increase in PI3K signalling by itself is not sufficient to trigger AVMs^28^. One could speculate that increased PI3K signalling leads to AVMs only in the context of reduced Smad or Notch signalling. Regardless of the precise mechanism, the data shown here demonstrate that blocking BMP9/10 signalling induces an increase in PI3K signalling and identify PI3K signalling pathway inhibition as a putative novel therapeutic approach for HHT2 patients.

PI3K signalling is activated by various growth factors, including VEGF^43^. Our data show that VEGF signalling is increased in *ALK1* mutant cells. Several possible causes for enhanced VEGF signalling exist: first, hypersprouting and AVM formation in BMP9/10-Alk1 signalling-deficient retinas probably leads to hypoxia, which is known to increase Vegf levels, and VEGF levels are also increased in human HHT patients^44^. Second, we and others have previously shown that BMP9 treatment leads to an increase in the expression of Notch signalling components Hey1 and Hey2, as well as in Vegfr1, and Notch and Vegfr1 counteract Vegf signalling through Vegfr2 in retina vessels^15^,^47^,^48^,^49^. Chromatin immunoprecipitation sequencing analysis had shown that Smad1,5 binding to the promoter of Hey1 and Hey2 was increased after BMP9 treatment of HUVECs^48^, suggesting that BMP9-Smad directly activates Notch signalling components. We and others^15^ also observe that *Alk1* mutant endothelial cells show a corresponding decrease in *Vegfr1* and Notch signalling components. Although we did not see effects on *Vegfr2* levels, Hey2 (HESR1) was previously shown to decrease VEGFR2 promoter-luciferase reporter activity^49^. Decreased VEGFR1 levels may be sufficient to account for the enhanced VEGF signalling shown in Fig. 2b; alternatively, VEGFR2 levels may change transiently at different time points.

We tested whether blocking excessive VEGF could rescue vascular defects in *Alk1* mutants. Administration of Vegf blAB in temporally inducible Alk1 mouse mutants has been shown to block progression of cutaneous AV shunts^50^. Furthermore, VEGF-blAB is currently tested in clinical trials in HHT patients^51^,^52^,^53^. We show that deletion of *Vegfr2*, the main signal transducing receptor for Vegf, prevents angiogenesis, but does not fully rescue normal vascular patterning and AVM formation. In fact, the severity of the vascular phenotype in *Alk1;Vegfr2*^*iΔEC*^ mice was Vegfr2 dose dependent. We found that *Cdh5*-CreERT2-mediated *Vegfr2* excision was highly variable between mice, as described previously^54^. Proper interpretation of the results thus required correlating Vegfr2 protein levels in mouse lungs to the retinal vascular phenotype in each mouse. Using this approach, we found that strong inhibition of Vegfr2 expression severely impaired the development of retinal vasculature in *Alk1* mutants, as previously shown on a wild-type background^54^. *Vegfr2* deletion completely abolished the hypervascular phenotype resulting from *Alk1* deletion, indicating that Vegf signalling inhibition is dominant and overrides lack of *Alk1* signalling. However, the retinas of the *Alk1;Vegfr2*^*iΔEC*^ pups still displayed AV shunts, suggesting that additional signalling components may be involved in AVM formation. This idea is also supported by *in vitro* studies with HUVECs and mLECs, where PI3K signalling is increased in the absence of exogenous VEGF treatment. Thus, the data support the existence of a specific disease-causing enhanced PI3K activity downstream of *Alk1* deficiency that is at least partially VEGF independent. Nevertheless, our data show that down-modulation of excessive Vegf signalling normalizes vasculature, supporting potential clinical benefits of anti-VEGF treatment in HHT2 patients.

Finally, our data confirm that BMP9/10 are physiological Alk1 ligands in mice. In previous studies, blocking of BMP10 in BMP9 mutants or ligand sequestration by Alk1-Fc treatment induced retina hypervascularization, but not AVM formation^45^,^47^, leaving open the possibility that additional Alk1 signalling mechanisms might protect from AVM formation. In these studies, treatments were administered to pups at P1, a stage when retina vascularization begins, and arteries and veins have not yet formed^55^. When receptor deletion and ligand blockade are done at P3, a stage when retina arteries and veins have formed and carry flow^56^, AVMs develop, suggesting that flow is required for AVM formation in mice^21^. Furthermore, subjecting endothelial cells to laminar shear stress potentiates signalling induced by low concentrations of BMP9 *in vitro*^21^. In zebrafish embryos, arresting flow prevents AVM formation in *Alk1* mutants^57^, demonstrating that flow is an evolutionary conserved trigger of AVM formation. AVMs always form between the larger arteries and veins close to the optic nerve that carry most blood flow, whereas less perfused vasculature towards the angiogenic front develops hypervascularization. Thus, existing data suggest that BMP9/10 signalling through Alk1 protects vasculature from hypervascularization in low flow conditions and at the same time prevents AVM development induced by higher flow. AVMs form via enhanced cell cycle progression and enlargement of capillary connections between retinal arteries and veins. Simply put, it appears that BMP9/10 signalling through Alk1 protects capillary size at vessel branch points, thereby allowing proper branching morphogenesis of the vasculature.

## Methods

### Mice

All animal experiments were performed under a protocol approved by Institutional Animal Care Use Committee of Yale University.

Eight weeks old *Alk1*^*iΔEC*^ and *Alk1;Vegfr2*^*iΔEC*^ mice of mixed genetic background were intercrossed for pups. Offsprings of both genders were used. Gene deletion was induced by intra-gastric injections with 50 μg Tx (Sigma, T5648; 1 mg ml^−1^) into *Alk1*^*iΔEC*^ and 100 μg Tx into *Alk1;Vegfr2*^*iΔEC*^ pups at P3 and P4. Tx-injected Cre-negative littermates were used as controls. BMP9/10 blABs (10 mg kg^−1^ per day) and the PI3K inhibitors were injected i.p.: BMP9/10 blABs between P2–P4, Wortmannin (0.4 mg kg^−1^ per day) or Pictilisib (20 mg kg^−1^ per day) once at P3, twice at P4 and once at P5.

### Latex dye injection

P5 pups were anaesthetized on ice, and abdominal and thoracic cavities were opened. The right atrium was cut, blood was washed out with 2 ml PBS and 1 ml of latex dye (Blue latex, BR80B; Connecticut Valley Biological Supply Co.) was slowly and steadily injected into the left ventricle with an insulin syringe. Retinas and GI tracts were washed in PBS and fixed with 4% paraformaldehyde (PFA) overnight. Retinas were stained with Isolectin B4 and GI tracts were cleared in 10% glycerol, 4 M urea and 0.1% Triton for 1–2 weeks before imaging.

### Reagents and antibodies

For immunodetection: anti-Alk1 (AF 770, 1 μg ml^−1^, R&D systems), anti-Eng (AF1320, 1 μg ml^−1^, R&D systems), anti-Jagged1 (AF599, 1 μg ml^−1^, R&D systems), anti-Vegfr1 (AF471, 2 μg ml^−1^, R&D systems), anti-Erg1/2/3 (SC353, 2 μg ml^−1^, Santa Cruz), anti-α-SMA CY3 (CY3-SMA, C6198, 1:200 working solution, Sigma), anti-phosphoS6 (5364, 1:200 working solution, Cell Signalling), anti-CD144 (555289, 2 μg ml^−1^, BD Pharmingen), IB4 (121412, 10 μg ml^−1^, Life Technologies), anti-pFOXO1 (9464, 1:100 working solution, Cell Signalling), anti-FOXO1 (2880, 1:100 working solution, Cell Signalling), anti-pPTEN (9549, 1:100 working solution, Cell Signalling), anti-PTEN (9188, 1:100 working solution, Cell Signalling) and Dapi (D1306, 1 μg ml^−1^, Life Technologies).

For western blotting: anti-Vegfr2 (9698, 1:2,000 working solution, Cell Signalling), anti-pVegfr2 (2478, 1:2,000 working solution, Cell Signalling), anti-pAKT (4060, 1:1,000 working solution, Cell Signalling), anti-AKT (4060, 1:1,000 working solution, Cell Signalling), anti-p-p44/42 mitogen-activated protein kinase (phospho-ERK, 9106, 1:2,000 working solution, Cell Signalling), anti-p44/42 mitogen-activated protein kinase (total ERK, 9102, 1:2,000 working solution, Cell Signalling), anti-pFOXO1 (9464, 1:1,000 working solution, Cell Signalling), anti-FOXO1 (2880, 1:1,000 working solution, Cell Signalling), anti-pPTEN (9549, 1:1,000 working solution, Cell Signalling) and anti-PTEN (9188, 1:1,000 working solution, Cell Signaling). Appropriate secondary antibodies were fluorescently labelled (Alexa Fluor donkey anti-rabbit, Alexa Fluor donkey anti-goat, Alexa Fluor donkey anti-rat, 1:400 working solution, Invitrogen) or conjugated to horseradish peroxidase (Anti-Rabbit IgG (H+L), 1:8,000 working solution, Vector Laboratories).

BMP9/10 blABs were from Genentech and PI3K inhibitors Wortmannin S2758 and Pictilisib S1065 were purchased from Selleckchem.

### Fluorescence-activated cell sorting

Retinas were harvested at P5, dissected in cold PBS and digested in 1 mg ml^−1^ Collagenase type II (Sigma) in DMEM (Sigma). Cells were incubated with CD31-APC (BD 551262, 2 ng ml^−1^) and CD45-FITC (11-0452-85, 5 ng ml^−1^, eBioscience), Hoechst (B2261, 25 μg ml^−1^, Sigma Aldrich) and Pyronin Y (P9172, 200 ng ml^−1^, Sigma Aldrich). CD31+/CD45− endothelial cells were isolated by FACS and further analysed for their cell cycle distribution by two-dimensional analysis of Hoechst and Pyronin Y fluorescence signal.

### mLECs isolation

MLECs were isolated from collagenase-digested lung tissue using rat-anti-mouse CD31 monoclonal antibody-coated Dynabeads (11035, Invitrogen). Primary mLECs were cultured in DMEM supplemented with 10% fetal bovine serum (FBS), 100 U ml^−1^ penicillin, 100 μg ml^−1^ streptomycin, 100 μg ml^−1^ endothelial cell mitogen (Biomedical Technologies, Inc.) and 10 U ml^−1^ heparin for 10 days.

### Immunostaining

The eyes of P5/P6 pups were prefixed in 4% PFA for 20 min at room temperature (rt). Retinas were dissected, blocked for 30 min at rt in blocking buffer (1% fetal bovine serum, 3% BSA, 0.5% Triton X-100, 0.01% Na deoxycholate, 0.02% Na azide in PBS at pH 7.4) and then incubated with specific antibodies (mentioned above) in blocking buffer overnight. The following day retinas were washed and then incubated with IB4 and corresponding secondary antibody for 1 h at rt and mounted in fluorescent mounting medium (DAKO, Carpinteria, CA, USA).

Endothelial cells in culture were fixed in 4% PFA for 15 min, permeabilized in 0.1% Triton X-100 in PBS for 10 min, blocked in 3% BSA (AB01088-00100, AmericanBIO) and incubated with the indicated primary antibodies overnight at 4 °C (mentioned above) followed by 1 h incubation with appropriate secondary antibodies at rt.

High-resolution pictures were acquired using a Leica SP5 confocal microscope with a Leica spectral detection system (Leica 15 SP detector) and the Leica application suite advanced fluorescence software. Quantification of retinal vasculature was done using ImageJ.

### Cell culture and siRNA transfection

HUVEC cells were obtained from the Yale University Vascular Biology and Therapeutics Core Facility and cultured in EGM2-Bullet kit medium (CC-3156 & CC-4176, Lonza). Depletion of *ALK1* was achieved by transfecting 25 pmol siRNA (Qiagen, mixture of S102659972 and S102758392) with Lipofectamine RNAiMax (Invitrogen), following the manufacturer's instructions. Transfection efficiency was assessed by western blotting and qPCR. Experiments were performed 48 h post transfection and results were compared with siRNA *Ctrl* (ON-TARGETplus Non-targeting Pool D-001810-10-05)

### Western blotting

Cells were lysed with Laemmli buffer including phosphatase and protease inhibitors (Thermo Scientific, 78420, 1862209). Twenty micrograms of proteins were separated on 4–15% Criterion precast gel (567-1084, Bio-Rad) and transferred on nitrocellulose membrane (Biorad). Western blottings were developed with chemiluminescence horseradish peroxidase substrate (Millipore, WBKLS0500) on a Luminescent image Analyser, ImageQuant LAS 4000 mini (GE Healthcare). Bands were quantified using ImageJ. See Supplementary Fig. 5 for the uncropped immunoblots.

### Quantitative real-time PCR

RNAs from HUVEC or from mLECs were purified using RNeasy-kit (Qiagen). One RNA was reverse transcribed using SuperScript III (Invitrogen) and quantitative PCR were assayed using the corresponding primers (Qiagen): MmAcvrl1 (QT00161434), MmEng (QT00148981), MmUnc5b (QT00167846), MmFlt1 (QT00096292), MmNotch1 (QT00156982), MmJag1 (QT00115703), MmEfnb2 (QT00139202) and MmEphB4 (QT00120295). The expression levels were normalized to MmActin (QT00095242) and MmCdh5 (QT00110467).

### Data availability

The authors declare that the data supporting the findings of this study are included within the article and its Supplementary Information files, or are available from the authors upon request.

## Additional information

**How to cite this article:** Ola, R. *et al*. PI3 kinase inhibition improves vascular malformations in mouse models of hereditary haemorrhagic telangiectasia. *Nat. Commun.* **7**, 13650 doi: 10.1038/ncomms13650 (2016).

**Publisher's note**: Springer Nature remains neutral with regard to jurisdictional claims in published maps and institutional affiliations.

## Supplementary Material

Supplementary Information Supplementary Figures 1-5.
