# Supplementary material for: PI3 kinase inhibition improves vascular malformations in mouse models of hereditary haemorrhagic telangiectasia
Source: Nat Commun. 2016 Nov 29;7:13650. doi: 10.1038/ncomms13650 (PMC5141347; doi:10.1038/ncomms13650)
Supplement: Supplementary Information — Supplementary Figures 1-5. [file ncomms13650-s1.pdf]

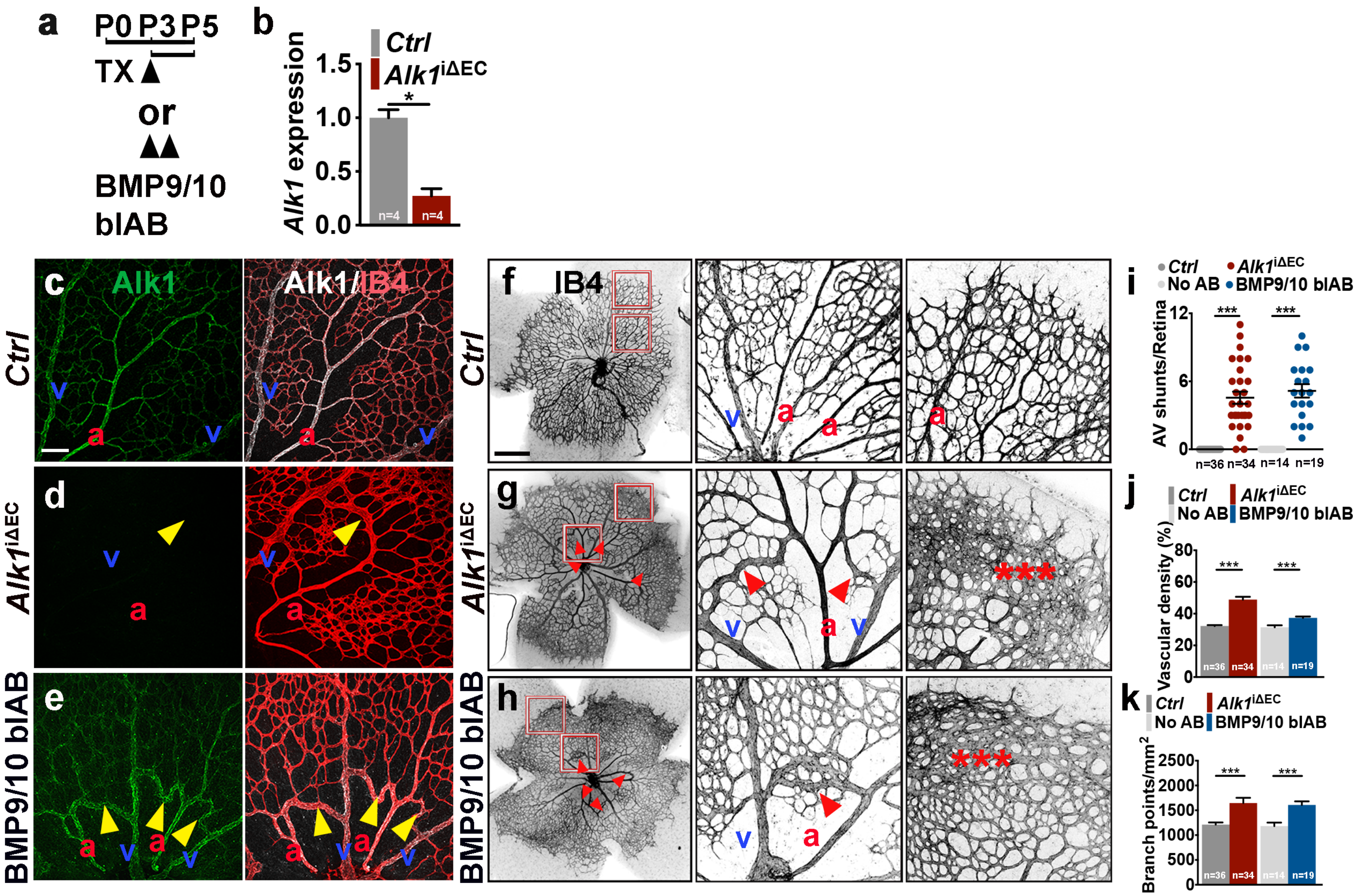

Supplementary Figure 1 . Blocking BMP9/10-Alk1 signalling induces vascular malformations.
a, Schematic of the experimental strategy to assess *Alk1* deletion or BMP9/10 blockade. Arrowheads indicate intra-gastric injection of 50µg Tx at postnatal day (P3) or the intraperitoneal injections of blocking antibodies (blAB) at P3 and P4. **b,** *Alk1* mRNA expression by qPCR using purified mouse lung endothelial cells (mLECs) isolated from P5 Tx injected mice. n=4 mice per group. Error bars: S.E.M, *P<0.05, student T test. **c-e,** IsolectinB4 (IB4) (red) and anti-Alk1 staining (green in single images and white in merged images) of retinal flat mounts from *Ctrl* (**c**), $Alk1^{i\Delta EC}$ (**d**) and BMP9/10 blAB injected P5 mice (**e**). Yellow arrowheads in **d** and **e** indicate retinal AV shunts. **f-h,** IB4 staining (negative images of the fluorescent signal) of retinal flat mounts from *Ctrl* (**f**), $Alk1^{i\Delta EC}$ (**g**) and BMP9/10 blAB injected P5 mice (**h**). Red arrowheads in **g** and **h** indicate retinal AV shunts and the red asterisks indicate vessel hypersprouting (middle and right panels are higher magnification of squared areas in the left panels). Scale bars: 100µm in **c**,**d**,**e** and 500µm in **f**,**g**,**h**. **i-k,** Quantification of AV shunt number, vascular density and number of branch points. n=14-36 retinas per group. Error bars: S.E.M, ***P<0.001, Mann-Whitney *U* test. **a**: artery in red, **v**: vein in blue.

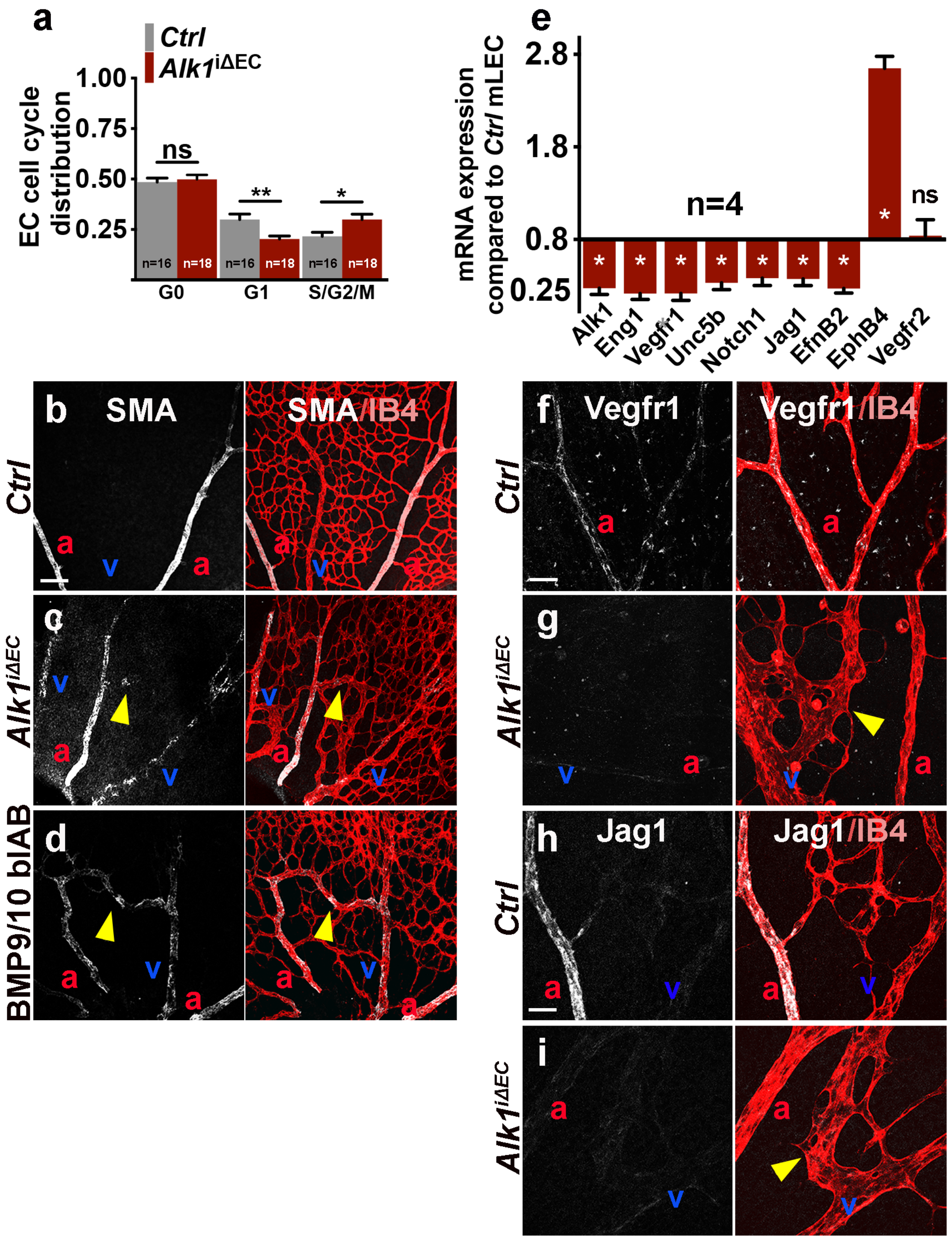

Supplementary Figure 2. Characterization of vascular malformations in mouse retinas.
a, Cell cycle analysis of retinal endothelial cells with the indicated genotypes. n=16-18 retinas per group. Error bars: S.E.M, ns- nonsignificant, *P<0.05, **P<0.01, Mann-Whitney *U* test. **b-d,** Smooth muscle actin (SMA) (white) and IsolectinB4 (IB4) (red) staining of retinal flat mounts from *Ctrl* (**b**), *Alk1$^{i\Delta EC}$* (**c**) and BMP9/10 blAB injected mice (**d**). **e,** qPCR analysis of mouse lung endothelial cells (mLEC) from *Alk1$^{i\Delta EC}$* compared to *Ctrl* P5 pups. n=4 mice per group. Error bars: S.E.M, *P<0.05, ns- non-significant, Student T test. **f-i,** Double labelling of retinal flat mounts from *Ctrl* (**f**,**h**) and *Alk1$^{i\Delta EC}$* P5 mice (**g**,**i**) with anti-Vegfr1 (white) (**f**,**g**) or anti-Jag1 (white) (**h**,**i**) and IB4 (red). Yellow arrowheads in **c**,**d**,**g**,**i** indicate AV shunts. Scale bars: 100μm. **a**: artery in red, **v**: vein in blue.

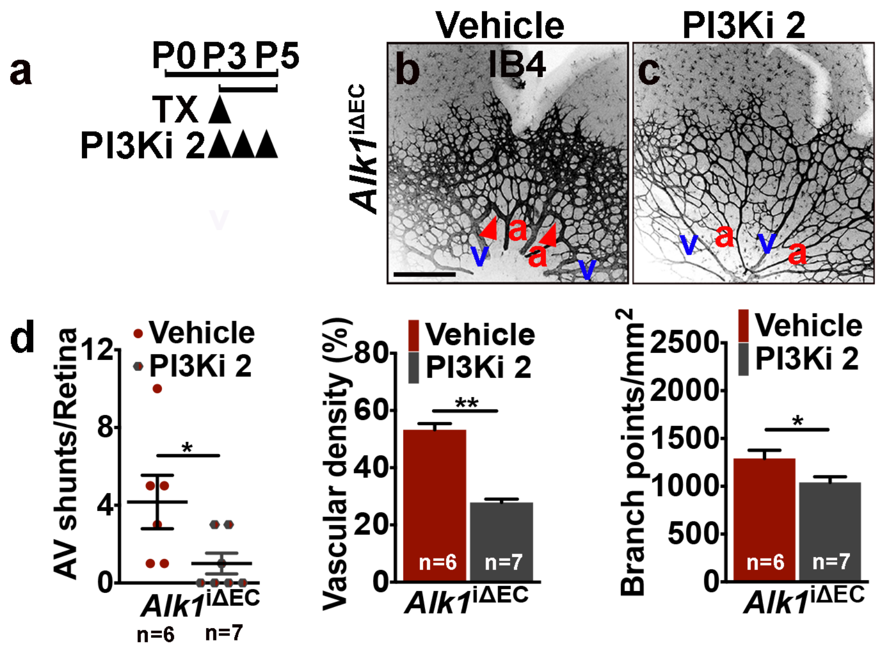

Supplementary Figure 3. Pictilisib -PI3K inhibitor prevents vascular malformations in *Alk1$^{i\Delta EC}$* retinas. a, Schematic representation of the experimental strategy to assess the effects of PI3K inhibition on *Alk1$^{i\Delta EC}$* retinal vasculature. Arrowheads indicate the time course of intra-gastric injection of 50µg Tamoxifen (Tx) at postnatal day 3 (P3) and the PI3K inhibitor-Pictilisib (intraperitoneal, PI3Ki 2, 20mg/kg) or Vehicle (DMSO) at P3-P5. **b-c,** IsolectinB4 (IB4) staining of retinal flat mounts (negative images of the fluorescent signal) from *Alk1$^{i\Delta EC}$* mice treated with vehicle or PI3Ki 2. Red arrowheads in **b** indicate AV shunts. **d,** Quantification of AV shunt number, vascular density and number of branchpoints. n=6-7 retinas per group. Scale bars: **b** and **c:** 200µm. Error bars: S.E.M, *P<0.05, **P<0.01, Mann-Whitney *U* test. **a**: artery in red, **v**: vein in blue.

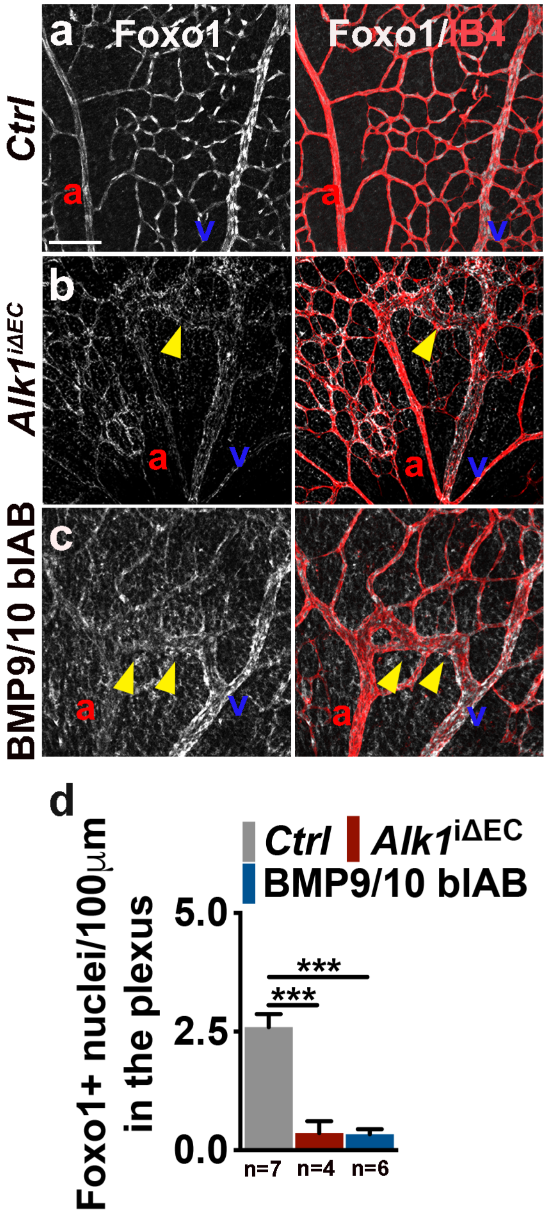

Supplementary Figure 4. Expression of PI3K signalling components. a-c, anti-Foxo1 (white) and IsolectinB4 (IB4) (red) staining of retinal flat mounts from *Ctrl* (**a**), *Alk1*$^{i\Delta EC}$ (**b**) and BMP9/10 bIAB injected P5 mice (**c**). Yellow arrowheads indicate retinal AV shunts in **b** and **c**. **d,** Quantification of IB4+/Foxo1+ nuclei per 100µm in the vascular plexus. n=4-7 retinas per group. Error bars: S.E.M, ***P<0.001, Mann-Whitney *U* test. Scale bars: 100µm. **a**: artery in red, **v**: vein in blue.

Supplementary Figure 5. Uncropped immunoblots

Uncropped blots for Figure 2b

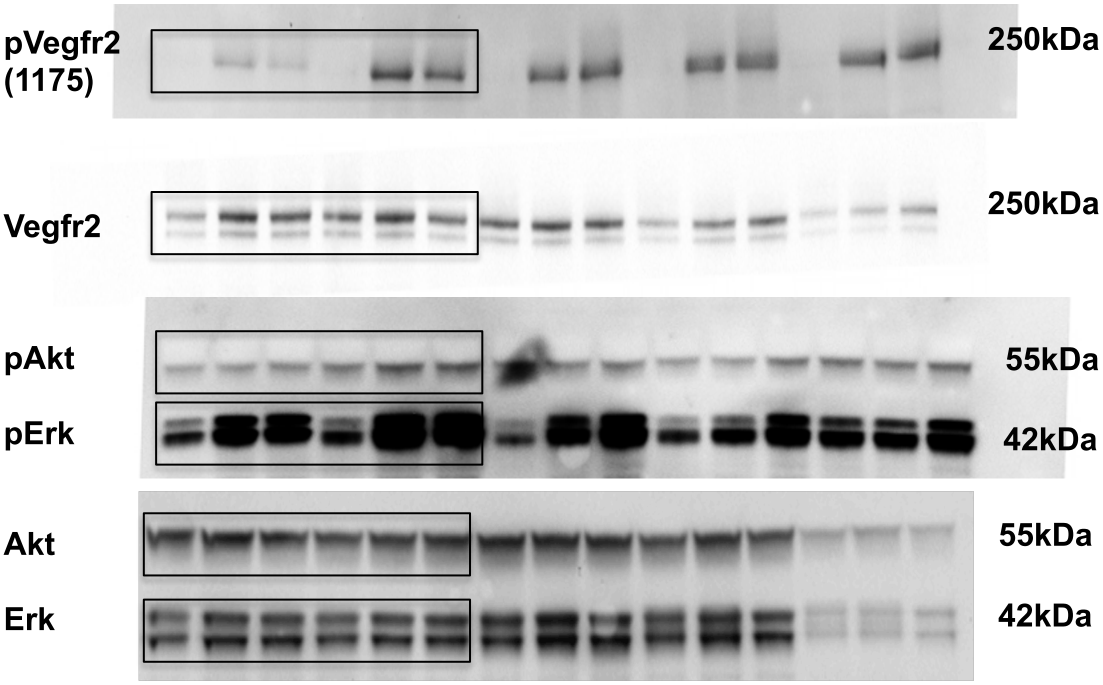

Uncropped blots for Figure 2j

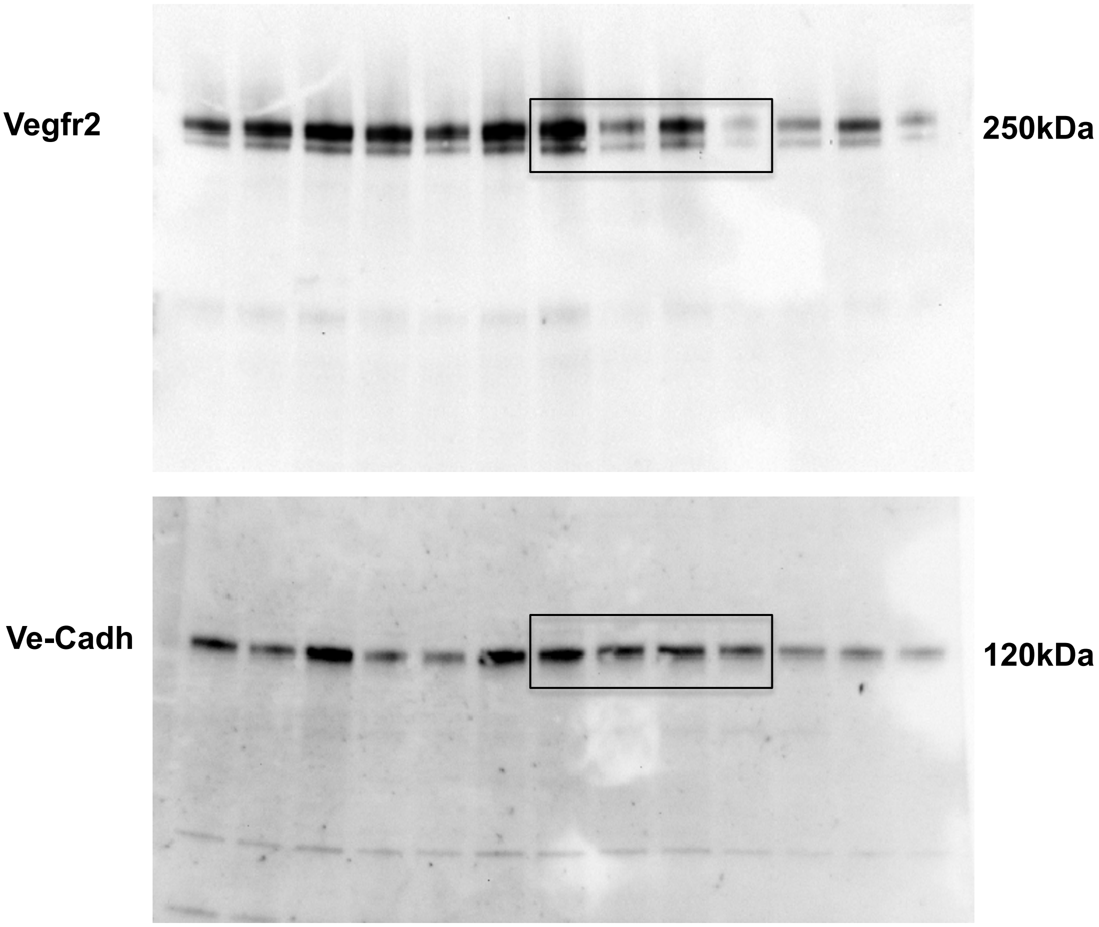

Uncropped blots for Figure 3a

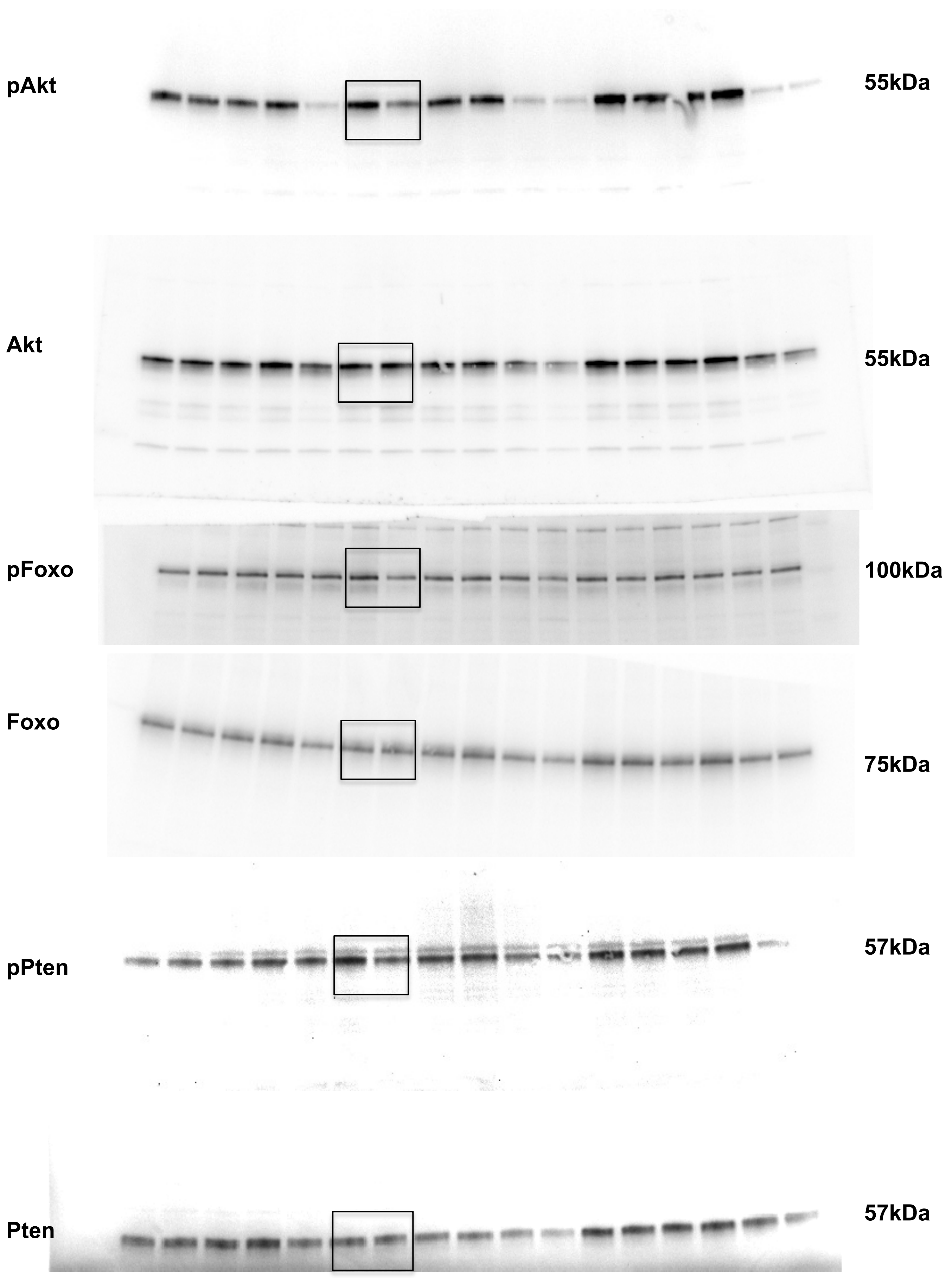

Uncropped blots for Figure 3c

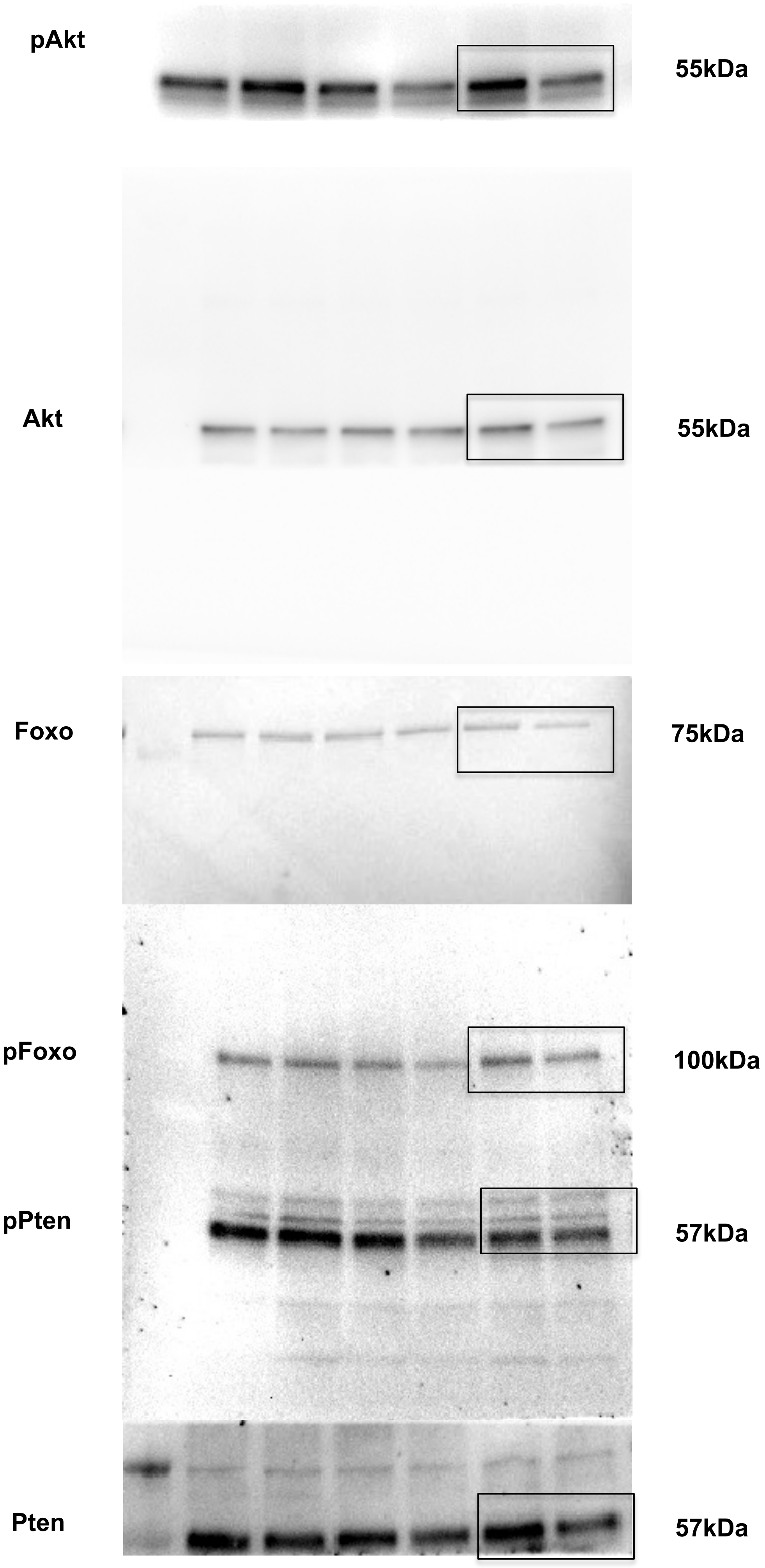

Uncropped blots for Figure 3e

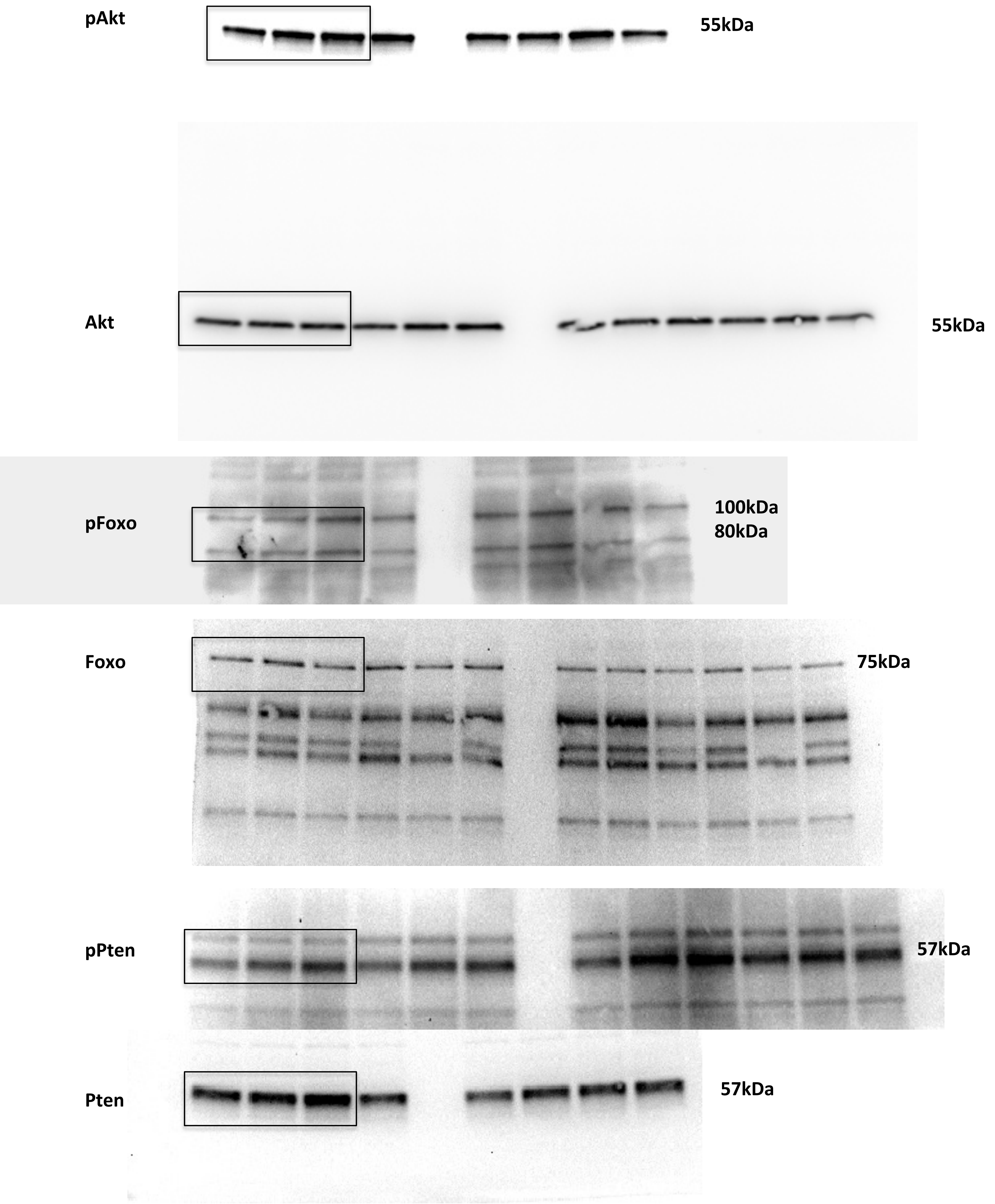

Uncropped blots for Figure 3g

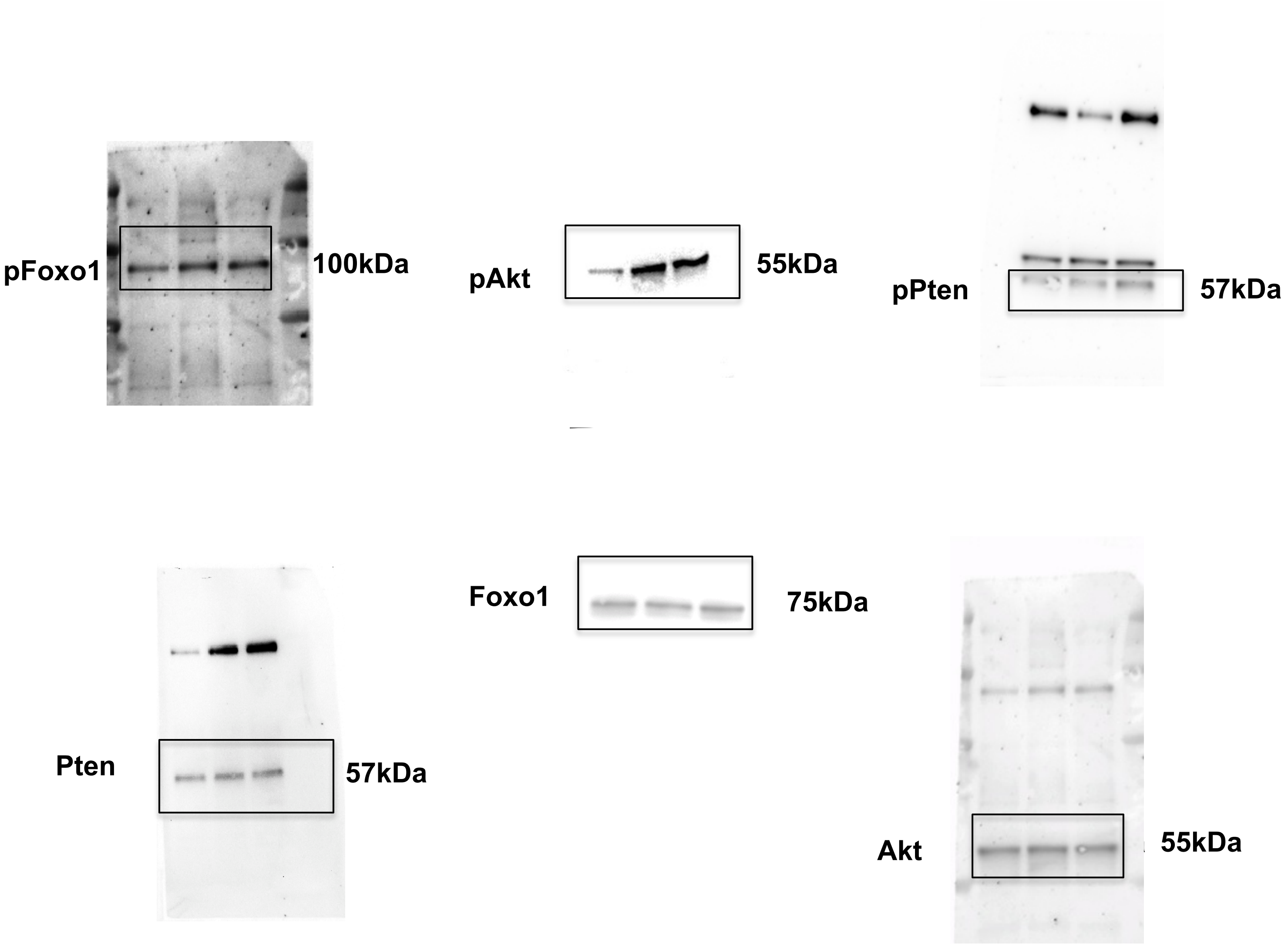

Uncropped blots for Figure 4b

pAkt

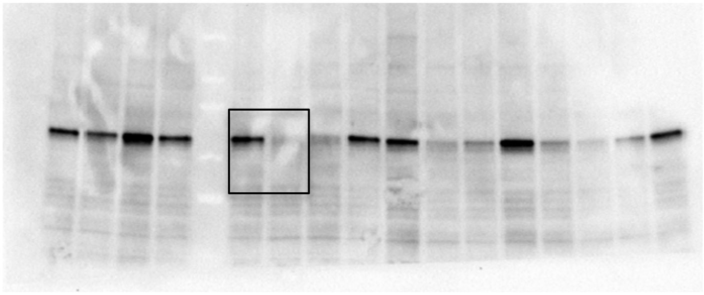

55kDa

Akt

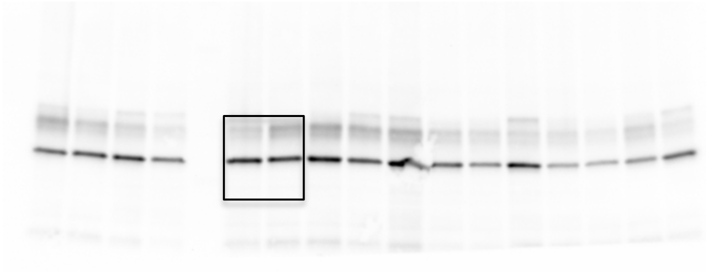

55kDa
